# 14-3-3 Proteins Interact with a Hybrid Prenyl-Phosphorylation Motif to Inhibit G Proteins

**DOI:** 10.1016/j.cell.2013.05.003

**Published:** 2013-05-23

**Authors:** Philippe Riou, Svend Kjær, Ritu Garg, Andrew Purkiss, Roger George, Robert J. Cain, Ganka Bineva, Nicolas Reymond, Brad McColl, Andrew J. Thompson, Nicola O’Reilly, Neil Q. McDonald, Peter J. Parker, Anne J. Ridley

(Cell *153*, 640–653; April 25, 2013)

In the original published version of the above article, the Protein Data Bank accession number for the 14-3-3ζ-Rnd3 peptide atomic coordinates and structure factors was inadvertently omitted. The number is 4BG6 and has been added to the article online. We apologize for any inconvenience.

