# Supplementary material for: 14-3-3 Proteins Interact with a Hybrid Prenyl-Phosphorylation Motif to Inhibit G Proteins
Source: Cell. 2013 Apr 25;153(3):640–53. doi: 10.1016/j.cell.2013.03.044 (PMC3690454; doi:10.1016/j.cell.2013.03.044)
Supplement: Document S1. Tables S1–S5 [file mmc1.pdf]

Table S1. Identification of 14-3-3 Proteins as Rnd3 Interactors by Mass Spectrometry, Related to Figure 1

protein I.D.	accession no.	MW (Da)	pI	no. peptides	unique peptides	% coverage
Rnd3	P61587	27351	8.78	14	14	63
14-3-3ε	P62258	29155	4.63	9	7	51
14-3-3γ	P61981	28285	4.8	9	4	37
14-3-3ζ	P63104	27728	4.73	6	3	33
14-3-3β	P31946	28065	4.76	5	3	24

FLAG-Rnd3 was expressed in COS7 cells. FLAG-Rnd3 immunoprecipitates were resolved by SDS-PAGE, and the corresponding bands excised from the gel and analysed by mass spectrometry. Endogenous 14-3-3 proteins, which migrate at a similar molecular weight to FLAG-Rnd3, were identified.

Table S2. 14-3-3β Inhibits Rnd3-Induced Morphological Changes, Related to Figure 3

comparison	p-value	statistical significance
mock : β	0.957	NS
mock : Rnd3WT	<0.001	***
mock : Rnd3WT+β	<0.001	***
mock : Rnd3A240	<0.001	***
mock : Rnd3A240+β	<0.001	***
β : Rnd3WT	<0.001	***
β : Rnd3WT+β	<0.001	***
β : Rnd3A240	<0.001	***
β : Rnd3A240+β	<0.001	***
Rnd3WT : Rnd3WT+β	<0.001	***
Rnd3WT : Rnd3A240	0.999	NS
Rnd3WT : Rnd3A240+β	0.999	NS
Rnd3WT+β : Rnd3A240	<0.001	***
Rnd3WT+β : Rnd3A240+β	<0.001	***
Rnd3A240 : Rnd3A240+β	0.991	NS

NIH3T3 cells were transfected with the indicated FLAG-Rnd3 and HA-14-3-3β constructs, and after 24 h were fixed and stained for F-actin, and with antibodies to FLAG and HA epitopes. Technical triplicates within 3 independent experiments were summed to give cell counts per experiment per phenotype per condition (n=300). A Poisson model (generalized linear model) was used to fit the data, allowing the number of counted cells to vary between the treatment, the phenotype observed, and the two-way interactions between these. A multiple pairwise comparison between the cells with a "normal" phenotype for the various conditions was then performed and corrected for multiple testing by Tukey's method. The p-values for comparisons were calculated. β, 14-3-3β; NS, not significant.

Table S3. Rnd3 Peptide Sequences, Related to Figure 5

peptide sequence	name	experiment used for
N-DLRKDKAK**S**C-C	-P-F	ITC
N-DLRKDKAK**pS**C-C	+P-F	ITC
N-DLRKDKAK**S**C(S-farnesyl)-C	-P+F	ITC
N-DLRKDKAK**pS**C(S-farnesyl)-C	+P+F	ITC + crystal structure
N-CDRSK**pS**APTS-C	PKC	peptide competition
N-Bio-Eahx-DLRKDKAK**S**C-C	-P-F	peptide competition & pull-down
N-Bio-Eahx-DLRKDKAK**pS**C-C	+P-F	peptide competition & pull-down
N-Bio-Eahx-DLRKDKAK**S**C(S-farnesyl)-C	-P+F	peptide competition & pull-down
N-Bio-Eahx-DLRKDKAK**pS**C(S-farnesyl)-C	+P+F	peptide competition & pull-down

Peptide sequences from Rnd3 C-terminus (last 10 amino acids of the protein sequence from amino (N-) to carboxy (-C) terminus) are shown in single letter amino acid code, with **pS** denoting phosphorylated serine. The farnesylation on C241 is indicated in brackets. Also shown is a PKCε peptide corresponding to a high affinity 14-3-3 binding sequence (Saurin et al., 2008), which was used as a positive control and inhibited the Rnd3/14-3-3 interaction. P, phosphorylated; F, farnesylated; Bio, biotinylated; ITC, isothermal titration calorimetry.

Table S4. X-Ray Data Collection and Refinement Statistics, Related to Figure 6

	**14-3-3/Rnd3 peptide**
**Data collection**	
Space group	$P2_12_12_1$
Cell dimensions	
a, *b*, *c* (/Å)	70.78, 81.31, 111.19
α, β, γ (°)	90, 90, 90
Resolution (/Å)	2.3
R_{merge} (%)	8.4 (53.9)
$I / \sigma I$	5.9 (1.2)
Completeness (%)	99.1 (99.1)
Multiplicity	3.21 (3.20)
Wilson B	53.3
Wavelength (/Å)	0.97
Detector Distance (/mm)	439
**Refinement**	
Resolution (/Å)	2.3
No. reflections (unique)	92928 (28921)
R_{work} / R_{free}	24.4 / 27.3
No. Atoms	
Protein	3652 (including Farnesyl)
Ligand/ion	63 (PEG)
Water	80
B-factors	
Protein	Chain A: 71.2, Chain B: 64.9, Chain Q: 88.5, Chain R: 83.4
Ligand/ion	Chain P (PEG): 70.2
Water	Chain W: 100.4
R.m.s. deviations	
Bond lengths (/Å)	0.001
Bond angles (°)	0.495

Table S5. Interactions of C-Terminal Peptides from Candidate Proteins with 14-3-3, Related to Figure 7

protein	peptide sequence	K_D (nM)	k_{on} ($M^{-1}s^{-1}$)	k_{off} (s^{-1})	χ^2	R^2
Rap1A	EKKKPKKK**pS**C-GG	23.6	1.4×10^6	3.4×10^{-2}	0.20	0.99
PDE6C	GGDDKKSK**pT**C-GG	44.5	4.1×10^5	1.8×10^{-2}	0.03	0.99
RPRG	TNTERRSK**pS**C-GG	95	2.4×10^5	2.2×10^{-2}	0.03	0.99
Rap1B	PGKARKKS**pS**C-GG	170	3.2×10^5	5.5×10^{-2}	0.01	0.99
RhoG	PTPIKRGR**pS**C-GG	868	4.1×10^4	3.5×10^{-2}	0.02	0.99

The affinities of the indicated phosphorylated and geranylgeranylated peptides for 14-3-3ζ were determined by biolayer interferometry using an Octet Red96 instrument. Data of a typical experiment ($n \geq 3$) along with statistics of the fit (χ^2, R^2) are shown. Peptide sequences from the C-terminus of the indicated proteins (last 10 amino acids of the mature protein sequence, terminating with Cys of CAAX box) are shown in single letter amino acid code, with **pS/pT** denoting phosphorylated serine/threonine. The modifications added to the C-terminal Cys are indicated. GG, geranylgeranylated; K_D, dissociation constant; k_{on}, association rate constant; k_{off}, dissocation rate constant.
