# Supplementary material for: 14-3-3 Proteins Interact with a Hybrid Prenyl-Phosphorylation Motif to Inhibit G Proteins
Source: Cell. 2013 Apr 25;153(3):640–53. doi: 10.1016/j.cell.2013.03.044 (PMC3690454; doi:10.1016/j.cell.2013.03.044)
Supplement: Document S2. Article plus Supplemental Information [file mmc2.pdf]

Cell

14-3-3 Proteins Interact with a Hybrid Prenyl-Phosphorylation Motif to Inhibit G Proteins

Philippe Riou,[1,4] Svend Kjær,[5,10] Ritu Garg,[1,10] Andrew Purkiss,[6] Roger George,[5] Robert J. Cain,[1] Ganka Bineva,[7] Nicolas Reymond,[1] Brad McColl,[1] Andrew J. Thompson,[3,8] Nicola O'Reilly,[7] Neil Q. McDonald,[6,9] Peter J. Parker,[2,4,*] and Anne J. Ridley[1,*]
[1]Randall Division of Cell and Molecular Biophysics, New Hunt's House, Guy's Campus
[2]Division of Cancer Studies, New Hunt's House, Guy's Campus
King's College London, London SE1 1UL, UK
[3]MRC Centre for Neurodegeneration Research, De Crespigny Park, King's College London, London SE5 8AF, UK
[4]Protein Phosphorylation Laboratory
[5]Protein Purification Facility
[6]Structural Biology Laboratory
[7]Peptide Synthesis Laboratory
Cancer Research UK London Research Institute, Lincoln's Inn Fields, London WC2A 3LY, UK
[8]The Institute of Cancer Research, Chester Beatty Laboratories, 237 Fulham Road, London SW3 6JB, UK
[9]Institute of Structural and Molecular Biology, Department of Biological Sciences, Malet Street, Birkbeck College, University of London, London WC1E 7HX, UK
[10]These authors contributed equally to this work
*Correspondence: peter.parker@cancer.org.uk (P.J.P.), anne.ridley@kcl.ac.uk (A.J.R.)
http://dx.doi.org/10.1016/j.cell.2013.03.044

SUMMARY

Signaling through G proteins normally involves conformational switching between GTP- and GDP-bound states. Several Rho GTPases are also regulated by RhoGDI binding and sequestering in the cytosol. Rnd proteins are atypical constitutively GTP-bound Rho proteins, whose regulation remains elusive. Here, we report a high-affinity 14-3-3-binding site at the C terminus of Rnd3 consisting of both the Cys241-farnesyl moiety and a Rho-associated coiled coil containing protein kinase (ROCK)-dependent Ser240 phosphorylation site. 14-3-3 binding to Rnd3 also involves phosphorylation of Ser218 by ROCK and/or Ser210 by protein kinase C (PKC). The crystal structure of a phosphorylated, farnesylated Rnd3 peptide with 14-3-3 reveals a hydrophobic groove in 14-3-3 proteins accommodating the farnesyl moiety. Functionally, 14-3-3 inhibits Rnd3-induced cell rounding by translocating it from the plasma membrane to the cytosol. Rnd1, Rnd2, and geranylgeranylated Rap1A interact similarly with 14-3-3. In contrast to the canonical GTP/GDP switch that regulates most Ras superfamily members, our results reveal an unprecedented mechanism for G protein inhibition by 14-3-3 proteins.

INTRODUCTION

Most Ras superfamily G proteins cycle between an inactive GDP-bound conformation and an active GTP-bound conformation, which signals to downstream targets to induce cellular responses. They are activated by guanine nucleotide exchange factors (GEFs) and inactivated by GTPase activating proteins (GAPs), which catalyze GTP hydrolysis. The three Rnd proteins, Rnd1, Rnd2, and Rnd3 (also known as RhoE) are a subfamily of the Rho GTPase family with atypical properties (Foster et al., 1996; Riou et al., 2010). They are constitutively GTP-bound because they have amino acid substitutions in key residues involved in GTP hydrolysis, and have a very low affinity for GDP. Their activity must therefore be regulated differently to classic G proteins (Riou et al., 2010). For Rnd3, one such mechanism is phosphorylation by Rho-associated coiled coil containing protein kinase (ROCK)1 and protein kinase C (PKC)α, which shifts Rnd3 subcellular localization from the plasma membrane to the cytoplasm and increases its stability (Madigan et al., 2009; Riento et al., 2005). The molecular basis for these effects remains uncharacterized. Rnd2 localizes predominantly to the cytoplasm, whereas Rnd1 is normally localized on membranes (Roberts et al., 2008). Whether the localization of Rnd1 and Rnd2 is also regulated by phosphorylation is not known.

Like most Ras superfamily G proteins, Rnd proteins are post-translationally polyisoprenylated on a Cys residue, four amino acids from the C terminus (Cys of the CAAX box motif, where C represents cysteine; A an aliphatic amino acid; and X any amino acid residue, which determines the type of isoprenyl

 ©2013 Elsevier Inc.

group). Isoprenylation is followed by proteolytic removal of the AAX amino acids and carboxymethylation of the polyisoprenylcysteine. These irreversible modifications mediate the interaction of the GTPases with membranes and are generally required for their biological functions. Basic residues near the C terminus of some GTPases such as Rac1 and K-Ras4B also contribute to their membrane localization (Hancock et al., 1990; Michaelson et al., 2001; van Hennik et al., 2003). The Rho GTPases RhoA, Rac1, and Cdc42 are posttranslationally modified by a 20-carbon geranylgeranyl lipid and are solubilized from membranes and sequestered in the cytosol in an inactive state by binding to RhoGDIs, which have a hydrophobic pocket that accommodates the geranylgeranyl group (Hoffman et al., 2000). In contrast, Rnd proteins are modified by a shorter 15-carbon farnesyl group (Foster et al., 1996; Roberts et al., 2008), and Rnd3 does not bind and therefore is not extracted from membranes by RhoGDIs (Forget et al., 2002). This implies the existence of an alternative mechanism for the Rnd proteins to localize in the cytosol.

Rnd1 and Rnd3 induce loss of stress fibers and cell rounding (hence the name Rnd) in a variety of cell types and can stimulate cell migration (Riou et al., 2010). One way in which Rnd proteins regulate cell morphology is by inhibiting the Rho/ROCK signaling pathway and hence antagonizing actomyosin contractility. Overexpression of Rnd1 and Rnd3 stimulates p190RhoGAP activity, which reduces the amount of GTP-bound RhoA and decreases stress fibers (Wennerberg et al., 2003). Rnd3 also inhibits actomyosin contractility at cell-cell contacts in epithelial cells during collective cell migration (Hidalgo-Carcedo et al., 2011).

Here, we identify 14-3-3 proteins as Rnd interaction partners. 14-3-3 proteins are regulatory molecules that bind many functionally diverse proteins, usually by interacting with Ser/Thr phosphorylated residues (Obsil and Obsilova, 2011). We show that 14-3-3 binds Rnd proteins through a phosphorylated Ser residue and the adjacent C-terminal farnesyl group. By solving the crystal structure of a C-terminal Rnd3 peptide/14-3-3 complex, we show that 14-3-3 proteins directly interact with the farnesyl group via a hydrophobic surface, revealing lipid binding to 14-3-3. Using a consensus motif based on the Rnd3 C-terminal sequence, we identify several proteins with potential to bind to 14-3-3 in a similar fashion to Rnd3 and show that among them geranylgeranylated Rap1A interacts with 14-3-3. Therefore, 14-3-3 proteins can act as solubilizing factors specifically for phosphorylated and prenylated proteins from the Ras superfamily, translocating them from their site-of-action—on membranes—to the cytosol and consequently inhibiting their function.

RESULTS

Rnd3 Interacts with 14-3-3 Proteins via C-Terminal Phosphorylation Sites

Rnd3 was identified in immunoprecipitates of 14-3-3ε (Michael Yaffe, personal communication), and four endogenous 14-3-3 isoforms (β, ε, γ, ζ) were identified as Rnd3-binding partners by mass spectrometry analysis (Table S1 available online). This is the first time a Ras superfamily G protein has been reported to bind to 14-3-3 proteins. Seven 14-3-3 isoforms are known in mammals (Morrison, 2009), and because all isoforms coimmunoprecipitated with Rnd3 (Figure S1A), no isoform specificity was observed.

In most cases, 14-3-3 proteins interact with phosphorylated Ser/Thr residues on their target proteins (Morrison, 2009). Treatment of cells with the general kinase inhibitor staurosporine reduced Rnd3/14-3-3 interaction, and conversely the phosphatase inhibitor calyculin A increased the interaction (Figure S1B). In addition, treatment of Rnd3 immunoprecipitates with calf-intestinal phosphatase to dephosphorylate Rnd3 abolished subsequent interaction with 14-3-3 proteins (Figure 1A). Rnd3 phosphorylation is therefore required for binding to 14-3-3.

Previously, we identified seven Ser/Thr residues in Rnd3 that can be phosphorylated in vitro by ROCK1, two near the N terminus and five near the C terminus (Riento et al., 2005; Figure 1B). PKC also phosphorylates Rnd3 on one or more of these sites (Madigan et al., 2009). Mutation of all seven sites to Ala (Rnd3-AllA; Figure 1B) prevented Rnd3 from binding to recombinant GST-14-3-3β in vitro or endogenous 14-3-3 proteins in cells (Figures S1C and S1E), indicating that at least one of these residues is involved in the interaction. Deletion of the Rnd3 C terminus, but not the N terminus, abolished 14-3-3 interaction (Figures S1D and S1E), implicating C-terminal residues specifically. To map which Rnd3 phosphorylation site(s) mediate 14-3-3 interaction, each Ser/Thr was individually mutated to Ala. Mutation of Rnd3 S240 alone totally prevented the interaction with 14-3-3 proteins, whereas mutation of either S210 or S218 reduced the interaction (Figure 1C). A putative phosphomimetic mutant, Rnd3-S240D, did not bind 14-3-3 proteins (Figure 1D), in accordance with previous studies showing that 14-3-3 proteins require phosphate groups and not simply negatively charged amino acid side chains for binding (Muslin et al., 1996; Obsil and Obsilova, 2011).

To determine whether any single phosphorylation site(s) was sufficient for 14-3-3 binding, each of the phosphorylation sites was reintroduced individually or in combination into the Rnd3-AllA background. Of these Rnd3 mutants, only Rnd3-AllA-S210+S240 and Rnd3-AllA-S218+S240 could interact similarly to wild-type (WT) Rnd3 (Figure 1E; data not shown), whereas Rnd3-AllA-S240 displayed a weak interaction with 14-3-3 proteins (Figure S1F). Taken together, this indicates that S240 phosphorylation is essential for interaction with 14-3-3 proteins and that either S210 or S218 phosphorylation is also required in combination with S240 for optimal binding. These three phosphorylation sites and surrounding amino acids are completely conserved in Rnd3 proteins from a range of species (Figure S1G; note that Rnd3 is only present in vertebrates [Boureux et al., 2007]), providing strong evidence that they play an important role in Rnd3 function.

C-Terminal Phosphorylation of Rnd3 by ROCK1 and PKC Leads to 14-3-3 Interaction

Both ROCK1 (but not ROCK2) and PKC are reported to phosphorylate Rnd3, although the site(s) phosphorylated by PKC have not been identified (Madigan et al., 2009; Riento et al., 2005). To determine which sites were phosphorylated by ROCK1 or PKC, Rnd3 phosphosite mutants were expressed in COS7 cells, and immunoprecipitated Rnd3 was incubated with ROCK1 kinase domain or PKCζ kinase domain (Figure 2A). ROCK1 phosphorylated each of the seven Ser/Thr

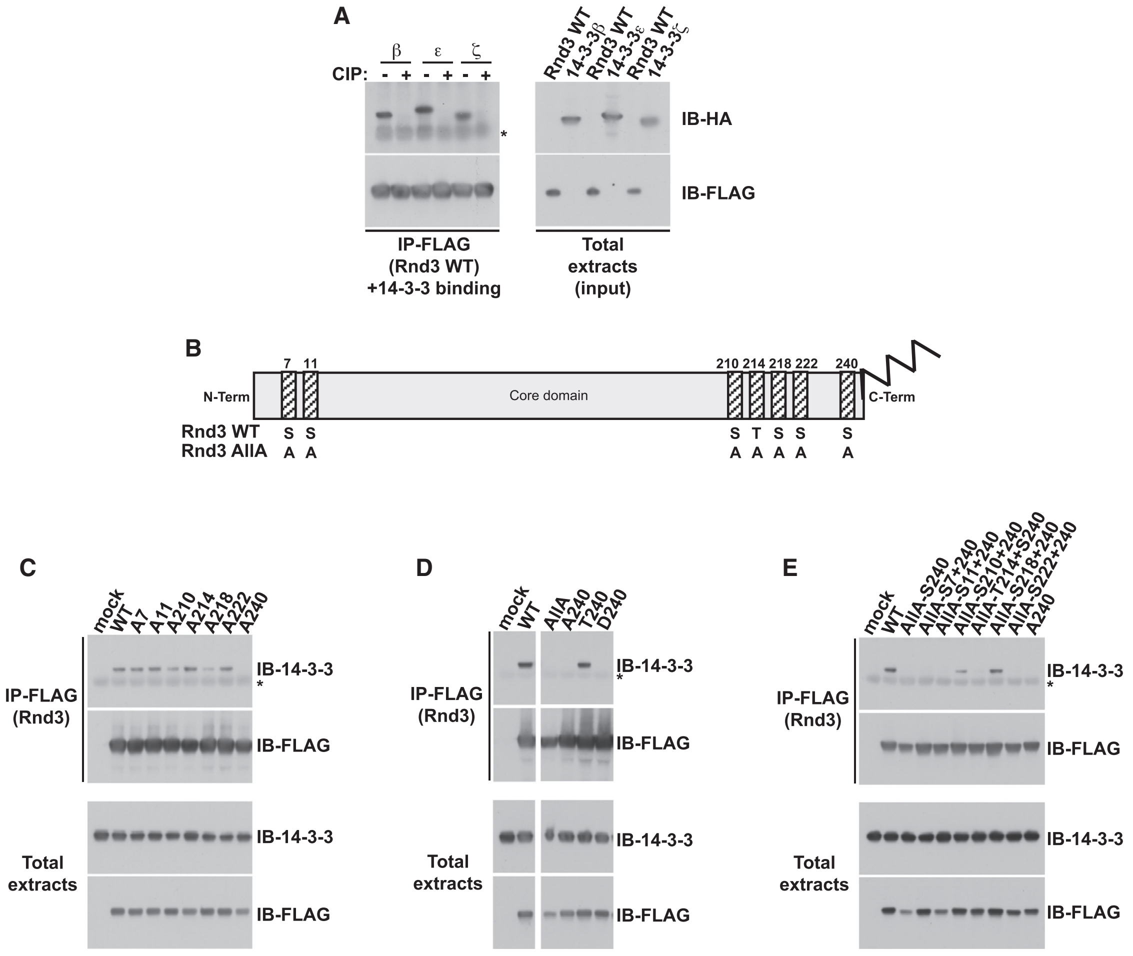

Figure 1. Rnd3 Binds to 14-3-3 Proteins via C-Terminal Phosphorylation Sites

(A) Expression vectors encoding FLAG-Rnd3 and HA-14-3-3 isoforms were transfected into COS7 cells. Cell lysates were immunoprecipitated (IP) with FLAG antibody, then treated with or without CIP for 1 hr. Immunoprecipitates were then incubated with cell lysates containing HA-14-3-3 proteins and immunoblotted (IB) with antibodies against FLAG and HA.

(B) Schematic showing 7 Rnd3 phosphorylation sites (S/T), mutated to A in AllA.

(C–E) The indicated FLAG-Rnd3 mutants were transfected into COS7 cells. Cells were lysed and immunoprecipitated with FLAG antibody. Endogenous 14-3-3 proteins bound to FLAG-Rnd3 were detected with 14-3-3 antibody. In (D), the space in the gel image marks the position of lanes that were not germane to these results and were thus removed during figure preparation for clarity.

Asterisk (*) indicates IgG light chain; S: Serine, T: Threonine, A: Alanine. See also Figure S1 and Table S1.

phosphorylation sites but S210 had a much lower level of phosphorylation than the other six sites. In contrast, only S210 was effectively phosphorylated by PKCζ. Hence, of the Rnd3 residues involved in 14-3-3 binding, S210 is a target for PKC, whereas both S218 and S240 are ROCK targets (Figure 2A). Note that PKC kinase domains have overlapping substrate specificity in vitro (Nishikawa et al., 1997), and S210 could potentially be phosphorylated by any PKC isoform, although PKCα seems to be the predominant isoform acting on Rnd3 in PMA-stimulated fibroblasts (Madigan et al., 2009). Interestingly, mutation of Ser240 to Thr did not affect 14-3-3 binding (Figure 1D), indicating that ROCK1 is likely to phosphorylate either Ser or Thr residues equally efficiently at this position.

To investigate the phosphorylation status of the three Rnd3 phosphorylation sites involved in 14-3-3 binding in cells, we first generated an antibody specific to pSer210 of Rnd3. Treatment of cells with PMA but not overexpression of ROCK1$^{1–420}$ stimulated an increase in pS210-Rnd3 and binding to 14-3-3, whereas the PKC inhibitor BIM1 reduced pS210-Rnd3, indicating that S210 is a PKC phosphorylation site in cells (Figure 2B). This antibody did not recognize other phosphorylation sites in Rnd3 (Figure 2C; data not shown). Mass spectrometry analysis of immunoprecipitated FLAG-Rnd3 was used to show that S218 and S240 were phosphorylated in cells expressing ROCK1$^{1–420}$ or treated with calyculin A (Figures S2A and S2B). Because S240 is adjacent to C241, which is normally farnesylated and methylated, we developed a methodology using synthetic peptides as references in order to be able to identify C-terminal modifications on Rnd3 immunoprecipitated from cell lysates. Rnd3-pS240 was specifically detected in a farnesylated, methylated peptide

(Figure S2B). As a control, clear identification of the farnesylated but not phosphorylated, C-terminal sequence was obtained for Rnd3-S240A (data not shown).

We also monitored Rnd3 phosphorylation in parallel to 14-3-3 binding using a pSer antibody raised against PKC substrate consensus sequences. Inhibition of PKC or ROCK decreased levels of Rnd3 phosphorylation detected with this pSer antibody, whereas in cells expressing constitutively active ROCK1$^{1–420}$ or stimulated with PMA, the levels increased (Figure 2B). This indicates that the antibody recognizes both ROCK1 and PKC phosphosites. Indeed, PKCs and ROCKs are both from the AGC family of kinases and have very similar consensus phosphorylation sites (Pearce et al., 2010). The pSer antibody binding to Rnd3 was strongly reduced by mutation of S210, S218, or S240, and similar to wild-type Rnd3 for the AllA-S210, S218, S240 mutant (Figure 2C), indicating that these are the major phosphosites that it recognizes. Similar results were observed using the Pro-Q Diamond reagent to measure Rnd3 phosphorylation (see Extended Experimental Procedures) (Figures 2B and 2C). These results together demonstrate for the first time that Rnd3 is phosphorylated in cells on S210 by PKC and on S218 and S240 by ROCK, leading to 14-3-3 binding (Figure S2C).

Rnd1 and Rnd2 Are Phosphorylated and Interact with 14-3-3 Proteins

Unlike Rnd3, phosphorylation of Rnd1 and Rnd2 has not been reported. Both Rnd1 and Rnd2 possess a Ser residue equivalent to Rnd3-S240 next to the CAAX box, as well as several other potential phosphorylation sites close to the C terminus (Figure 2D). These sites, however, are not conserved between the Rnd isoforms. We detected phosphorylation of Rnd1 and Rnd2 in cells, which was increased following addition of phosphatase inhibitor (calyculin A) to cells prior to lysis (Figure 2E). Under these conditions, and correlating with their level of phosphorylation, Rnd1 and Rnd2 were able to interact with 14-3-3 proteins (Figure 2E). Mutation of S228 in Rnd1 and S223 in Rnd2, both next to the CAAX box (Figure 2D), prevented 14-3-3 binding (Figure 2F). This suggests that Rnd1 and Rnd2 are regulated similarly to Rnd3 by C-terminal phosphorylation and 14-3-3 binding.

14-3-3 Binding Inhibits Rnd3 Function by Inducing Its Translocation from the Plasma Membrane to the Cytosol

Exogenous expression of Rnd3 typically induces cell rounding with long thin protrusions due to inhibition of Rho activity and subsequent loss of stress fibers and focal adhesions (Guasch et al., 1998; Nobes et al., 1998). This response is mediated in part by the interaction of Rnd3 with p190RhoGAP and stimulation of its GAP activity toward RhoA (Wennerberg et al., 2003). The Rnd3/14-3-3 interaction is not necessary for Rnd3-induced cell rounding because Rnd3-AllA, and other Rnd3 mutants defective in their ability to bind 14-3-3 proteins, induced a similar rounding response to wild-type Rnd3 in NIH 3T3 fibroblasts (Figure S3A). Both wild-type and Rnd3 phosphomutants localized to the plasma membrane in all cells with a rounded phenotype.

Rnd3-AllA coimmunoprecipitated with p190RhoGAP-B, similar to WT Rnd3 (Figure S3B), and thus 14-3-3 binding is not required for this interaction. As a control, Rnd3-T55V did not interact with p190RhoGAP-B (Figure S3B), consistent with previous data (Pacary et al., 2011; Wennerberg et al., 2003). Interestingly, Rnd3/14-3-3 and Rnd3/p190RhoGAP-B were in two mutually exclusive complexes: immunoprecipitates of p190RhoGAP-B contained Rnd3 but not 14-3-3 proteins, and conversely immunoprecipitates of 14-3-3 proteins contained Rnd3 but not p190RhoGAP-B (Figure S3C). In addition, Rnd3 bound to 14-3-3 proteins was phosphorylated on S210, whereas Rnd3 bound to p190RhoGAP-B was not (Figure S3C). This suggests that phosphorylation of Rnd3 increases its interaction with 14-3-3 proteins and reduces interaction with the effector p190RhoGAP.

We next investigated whether 14-3-3 binding inhibited Rnd3 function. As described above, Rnd3 and Rnd3-A240 both induced loss of stress fibers and cell rounding (Figures 3A, and S3A). Coexpression of 14-3-3β significantly inhibited the rounding response induced by wild-type Rnd3, but not Rnd3-A240, whereas 14-3-3β expression alone did not alter cell morphology (Figures 3A, 3B, and S3D; Table S2). By contrast, a mutant 14-3-3 protein that is defective for substrate binding (Thorson et al., 1998) did not inhibit Rnd3-induced cell rounding (data not shown). This provides evidence that the effect of 14-3-3β on cell morphology is specific to binding and inhibition of Rnd3 and not due to binding to another cellular target. Because 14-3-3 proteins bind to the C-terminal region of Rnd3 close to the farnesylation site (Figure S2C), we hypothesized that 14-3-3 binding could affect Rnd3 subcellular localization and consequently negatively regulate Rnd3 function. Indeed, as observed by immunofluorescence, 14-3-3β overexpression dramatically reduced the localization of Rnd3 but not Rnd3-A240 at the plasma membrane (Figure 3B). Importantly, the rounding response to WT Rnd3 and Rnd3-S240A correlated with Rnd3 localization at the plasma membrane (Figures 3B, S3A, and S3E). Similar to Rnd3, 14-3-3 overexpression inhibited Rnd1- and Rnd2-induced morphological changes, indicating that the function of all 3 Rnd proteins can be inhibited by 14-3-3 interaction (Figure S3F).

To confirm the effect of 14-3-3 proteins on Rnd3 localization, we investigated Rnd3 association with membranes by biochemical fractionation. Rnd3 was equally distributed between the cytosolic and membrane fractions but absent from the nuclear fraction. Coexpression of 14-3-3β and/or ROCK1 increased the proportion of Rnd3 in the cytosolic fraction, concomitant with reduced levels of Rnd3 in the membrane fraction (Figure 3C, upper left). In contrast, Rnd3-S240A localization was unaffected by ROCK1 or 14-3-3β expression (Figure 3C, upper right). 14-3-3 proteins localized to all fractions (Figure 3C, lower), as expected given their large number of cellular partners (Morrison, 2009). As a control, a Rnd3-C241S mutant was tested. C241 is the site of farnesyl group addition to Rnd3 (Figure S2C). Rnd3-C241S could not bind to membranes and localized only in the cytosolic fraction, as expected (Figure 3C, lower). Additional controls using PMA or calyculin A to increase Rnd3 phosphorylation or staurosporine to reduce phosphorylation showed that unphosphorylated Rnd3 mainly localizes to the membrane fraction, whereas phosphorylated Rnd3 accumulates in the cytosol (Figure 3C, lower panels). In addition, pS210-Rnd3 localized only in the cytosolic fraction and not the membrane fraction (Figure S3G), supporting our data indicating that Rnd3

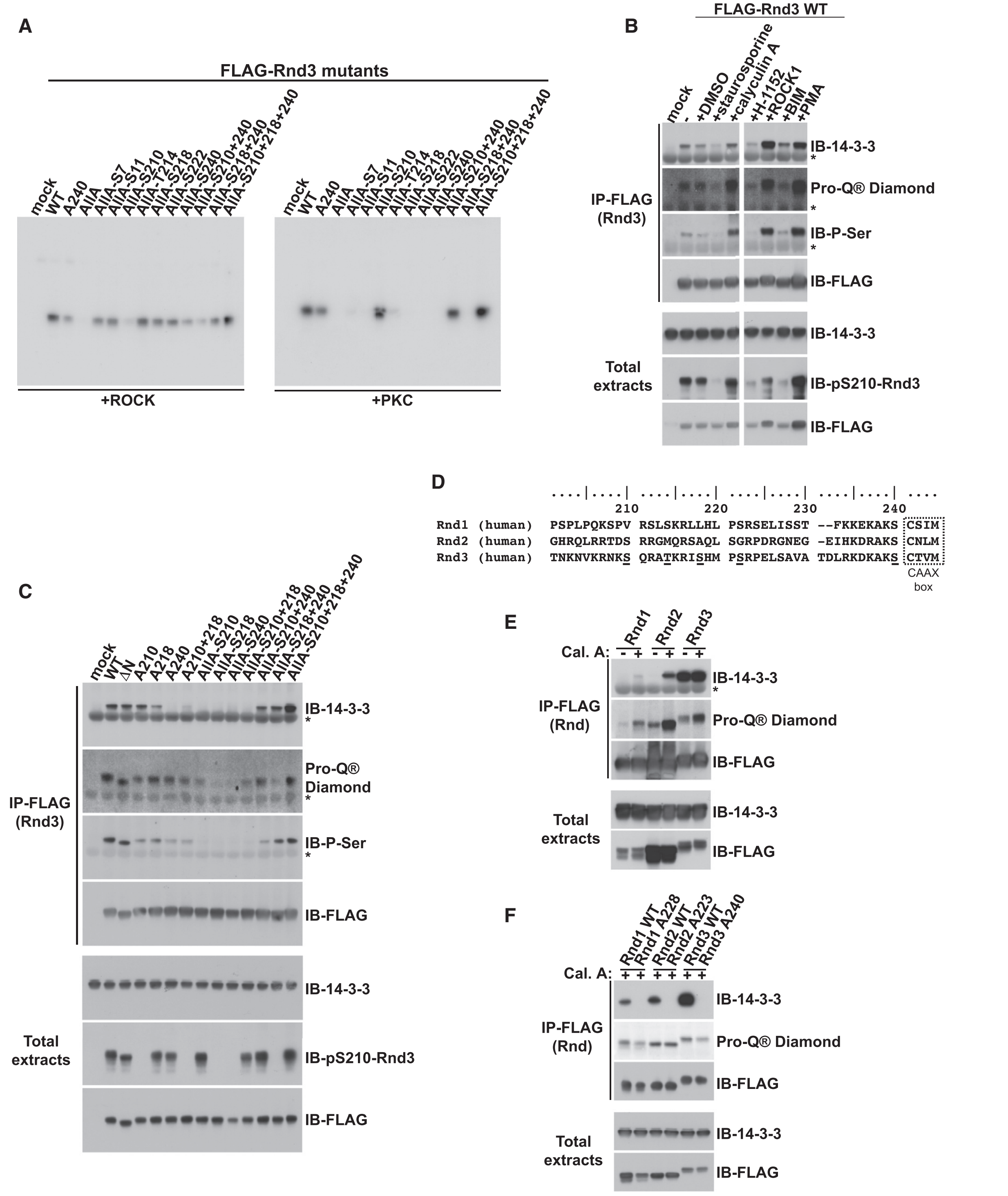

Figure 2. Rnd3 C-Terminal Phosphorylation by ROCK and PKC Is Required for 14-3-3 Interaction

(A–C) The indicated constructs were transfected into COS7 cells. Cell lysates were immunoprecipitated with FLAG antibody. (A) Immunoprecipitates were subjected to an in vitro kinase assay with either ROCK1 or PKCζ kinase domains. (B) FLAG-Rnd3 was co-overexpressed with ROCK1$^{1-420}$ where indicated. Cells were treated with chemical inhibitors (staurosporine, calyculin A, H-1152 [ROCK], BIM1 [PKC]) or the chemical activator PMA (PKC) for 2 hr (20 min for calyculin A) prior to lysis. Cell lysates were stained with Pro-Q Diamond reagent or immunoblotted with the indicated antibodies. The space in the gel image marks the position of lanes that were not germane to these results and were thus removed during figure preparation for clarity. (C) Endogenous 14-3-3 proteins were detected with anti-14-3-3 antibody. Membranes were also stained with Pro-Q Diamond reagent or immunoblotted with the indicated antibodies.

(legend continued on next page)

phosphorylation induces translocation from the membrane to the cytosol through 14-3-3 binding.

Taken together, these results indicate that plasma membrane-localized Rnd3 is active, binds p190RhoGAP and induces cell rounding and thin protrusions, whereas upon phosphorylation followed by subsequent 14-3-3 binding, it translocates to the cytosol where it is inactive and cannot alter cell morphology.

Rnd Farnesylation Is Required for 14-3-3 Interaction

The proximity of Rnd3 pS240 to farnesylated C241 (Figure S2C) led us to investigate the influence of farnesylation on 14-3-3 binding. We therefore mutated C241 to either G or S, or deleted the last four amino acids (Rnd3ΔCAAX), thus preventing Rnd3 farnesylation. Strikingly, none of the three CAAX box mutants interacted with 14-3-3 proteins (Figure 4A). Rnd3-C241S did not localize to the plasma membrane but in the cytoplasm and nucleus (Figures S4A and 3C), as expected in the absence of farnesylation (Roberts et al., 2008). Rnd3ΔC, which lacks the C-terminal phosphorylation sites and the CAAX box (Figure S1D), as expected, did not bind to 14-3-3 proteins (Figure 4A). Addition of a CAAX box to Rnd3ΔC did not restore 14-3-3 protein binding (Figure 4A), which is consistent with the requirement for the Rnd3 C-terminal phosphorylation sites for 14-3-3 interaction (Figures 1 and 2). As the interaction between Rnd3 and 14-3-3 proteins requires Rnd3 phosphorylation, the above data could be explained by the fact that Rnd3 needs to be at the plasma membrane, where it can be phosphorylated by the relevant kinases, and only then capable of binding 14-3-3 proteins. However, analysis of Rnd3 phosphorylation status revealed that this is unlikely because the different Rnd3 CAAX box mutants were still phosphorylated to comparable levels as the wild-type Rnd3 (Pro-Q Diamond, pS210; Figure 4A).

To validate the contribution of Rnd3 farnesylation to 14-3-3 interaction, we used a farnesyltransferase inhibitor (FTI). FTI treatment led to Rnd3 accumulation in the cytoplasm and nucleus (Figure S4A), as previously described (Roberts et al., 2008). FTI treatment prevented wild-type Rnd3 and Rnd3-AllA-S210/218/240 binding to 14-3-3 proteins (Figure 4B). FTI treatment only slightly reduced Rnd3 phosphorylation (ProQ Diamond, pS210; Figure 4B). Increasing the level of Rnd3 phosphorylation by coexpression of ROCK1$^{1–420}$ or treatment with PMA did not induce 14-3-3 interaction in the presence of FTI (Figure S4B). FTI treatment similarly inhibited Rnd1 and Rnd2 interaction with 14-3-3 (Figure 4C) and morphological changes (data not shown). These results indicate that Rnd farnesylation is specifically required for 14-3-3 protein interaction.

Phosphorylation of Ser-240 and Farnesylation of Cys-241 Are Both Required for Stable Rnd3 Binding to 14-3-3

Our data show that, in addition to phosphorylation, C-terminal farnesylation of Rnd proteins is required for their interaction with 14-3-3. To further investigate the role of each of these post-translational modifications to the Rnd3/14-3-3 interaction, we synthesized peptides corresponding to the last C-terminal ten amino acids of Rnd3 (232–241) that were phosphorylated on S240 and/or farnesylated at C241 (Table S3). Peptide competition experiments showed that, of the four peptides tested, the peptide that was phosphorylated but not farnesylated only slightly inhibited the interaction between GST-14-3-3β and Rnd3, whereas the peptide that was both phosphorylated and farnesylated strongly inhibited the interaction, indicating that farnesylation increases peptide binding to 14-3-3β (Figure 5A). Because the peptides were also biotinylated at the N terminus, we used a peptide pull-down assay to show that only the phosphorylated and farnesylated peptide could efficiently interact directly with recombinant 14-3-3β (Figure 5B). To further characterize the relative contributions of phosphorylation and farnesylation to binding, the affinity of each peptide for 14-3-3 proteins was determined by isothermal titration calorimetry. The farnesylated and phosphorylated peptide had an ~15-fold higher affinity for 14-3-3ζ compared to a phosphorylated, but not farnesylated, peptide (Table 1 and Figure 5C). The other two peptides did not bind detectably (Figure S5). These results indicate that both phosphorylation and farnesylation of Rnd3 are required for optimal binding to 14-3-3 and that the farnesyl group must directly interact with 14-3-3 proteins to stabilize the interaction.

Recognition of a Phosphorylated and Farnesylated Rnd3 C-Terminal Peptide by 14-3-3

To establish the structural basis for the requirement of both a farnesyl and phosphorylation modification for the 14-3-3:Rnd3 complex formation, we determined the 2.3 Å crystal structure of a Rnd3 farnesylated phosphorylated C-terminal peptide (spanning residues 232 to 241) bound to 14-3-3ζ (Table S4; Figure S6). Although the entire 15-carbon farnesyl moiety is not resolved in the structure, continuous electron density for the first two 5-carbon isoprenyl units (labeled as Pr1 and Pr2) is clearly evident extending from the Cys241 thiol and makes contacts to a hydrophobic patch within 14-3-3 (Pro165, Ile166, Leu216, Ile217, Leu220, and Leu172) (Figures 6A and 6B). The crystal structure shows how farnesylation extends the length of the Rnd3 C terminus able to interact with 14-3-3, despite Rnd3 lacking a consensus 14-3-3-binding motif flanking pSer240 at the C terminus (Figure 6C). This extension adds a significant hydrophobic surface to the interaction by engaging a complementary groove on 14-3-3 bearing hydrophobic residues. Other Rnd3 peptide contacts, more typical of high-affinity phosphodependent 14-3-3-binding motifs, include electrostatic interactions centered on pSer240 of Rnd3 and a basic cluster (Lys49, Arg56, and Arg127) on 14-3-3 (Figure 6B). Another important feature is the multiple hydrogen bonds made by main-chain atoms from Rnd3 with 14-3-3 side chains (Asn173, Asn224, and Glu180), serving to orient the backbone of the Rnd3 C terminus.

(D) Alignment of C-terminal regions of three human Rnd proteins, using Rnd3 protein sequence numbering. Sequences are shown in single letter amino acid code, with underlined S/T denoting known phosphorylated residues in Rnd3.

(E and F) COS7 cells transfected with the indicated constructs encoding wild-type or mutated Rnd1, Rnd2, or Rnd3 were treated with Calyculin A (Cal. A) for 20 min. Cell lysates were immunoprecipitated with FLAG antibody and stained with Pro-Q Diamond reagent or immunoblotted with antibodies to FLAG and 14-3-3. Asterisk (*) indicates IgG light chain. See also Figure S2.

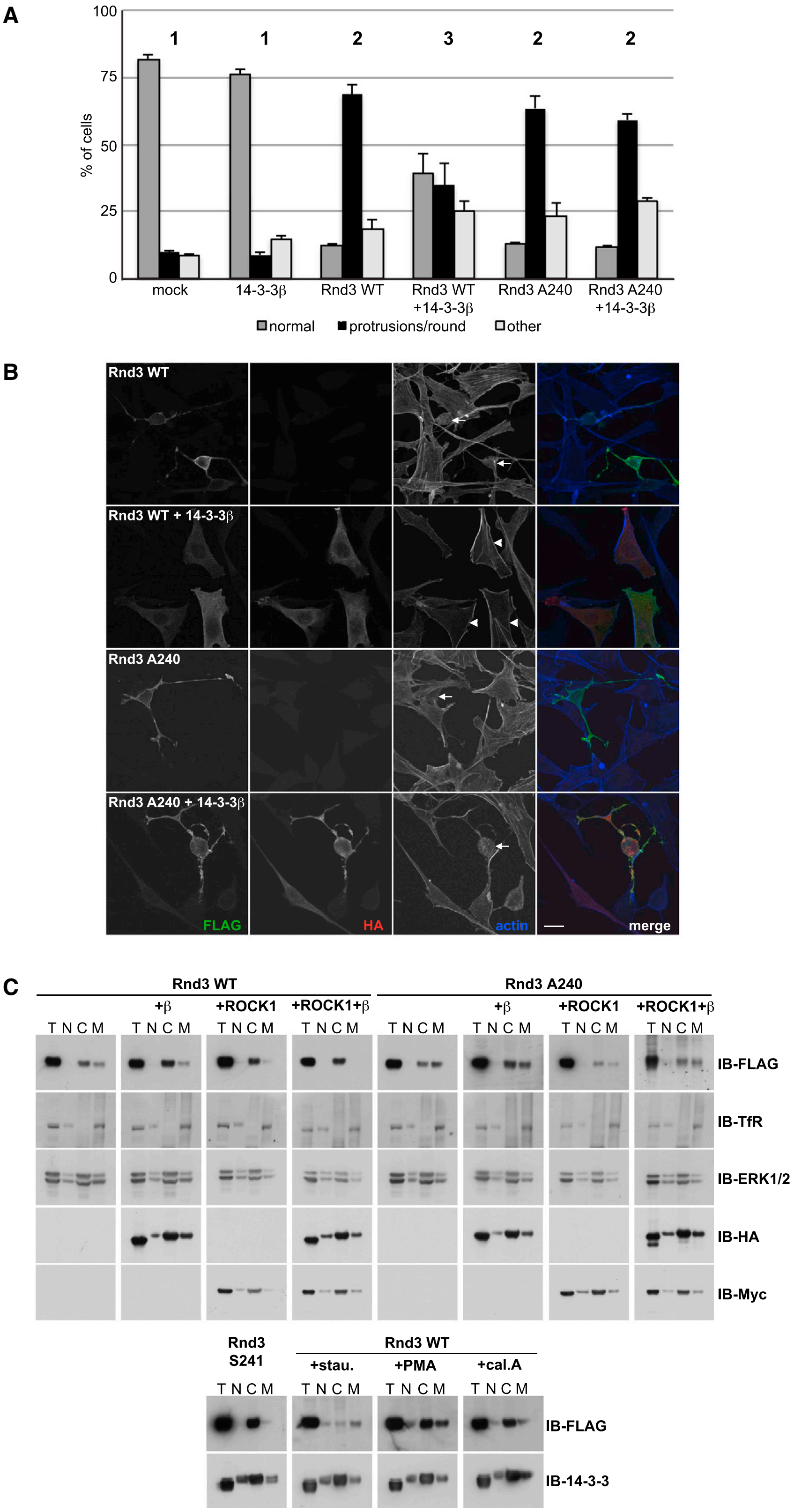

Figure 3. 14-3-3 Binding Inhibits Rnd3 by Inducing Translocation from the Plasma Membrane to the Cytosol

(A and B) NIH 3T3 cells were cotransfected with the indicated constructs. (A) Cell morphology quantification. Graph shows pooled results from technical triplicates in three independent experiments (n = 300 cells/condition/experiment). Conditions with the same number (1, 2, 3) are not statistically significantly different from each other. Error bars indicate mean ± SEM; Table S2 shows statistical analysis between the conditions. (B) Single confocal images (maximum intensity z stack projections in merge) showing cell morphology and FLAG-Rnd3 localization. Arrows: FLAG-Rnd3-expressing cells (F-actin images) with a rounded phenotype with protrusions. Arrowheads: normal phenotype. Scale bar, 20 μm.

(C) The indicated constructs were transfected into COS7 cells prior to cell lysis and biochemical fractionation. Fractions were immunoblotted with indicated antibodies. ERK1/2 and transferrin receptor are markers of cytosolic and membrane fractions, respectively. As controls (bottom panels), cells were transfected with the Rnd3 nonisoprenylated mutant S241 or Rnd3 WT with staurosporine (stau.), PMA, or calyculin A (cal. A) treatment. β, 14-3-3β; ROCK1, myc-ROCK1$^{1\text{-}420}$; T, total extracts; N, nuclear fraction; C, cytosolic fraction; M, membrane fraction. See also Figure S3 and Table S2.

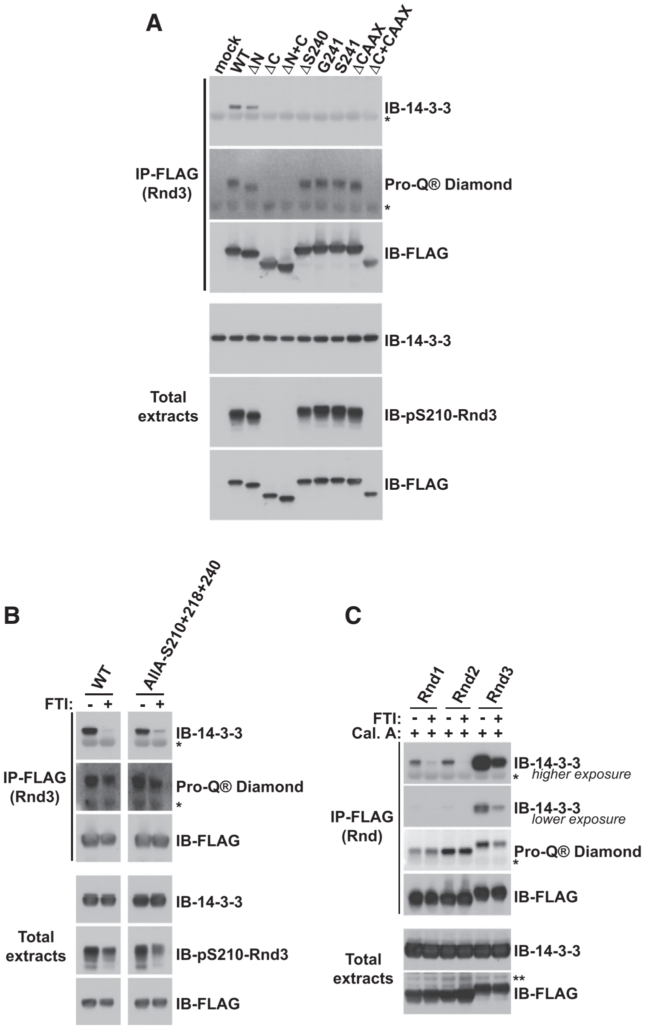

Figure 4. Rnd Farnesylation Is Required for Interaction with 14-3-3 Proteins

(A–C) The indicated constructs were transfected into COS7 cells. Cell lysates were immunoprecipitated with FLAG antibody. Endogenous 14-3-3 proteins bound to FLAG-Rnd3 were detected with 14-3-3 antibody. Membranes were stained with Pro-Q Diamond reagent or probed with the indicated antibodies. (B) COS7 cells were treated ± FTI overnight prior to cell lysis. The space in the gel image marks the position of lanes that were not germane to these results and were thus removed during figure preparation for clarity. (C) COS7 cells were treated with calyculin A (Cal. A) for 20 min ± FTI overnight prior to cell lysis. Asterisk (*) indicates IgG light chain. See also Figure S4.

The structure explains the requirement for the Rnd3 farnesyl group shown by pull-down and calorimetry data. The farnesyl moiety contributes significant additional hydrophobic contacts, effectively switching Rnd3 from having a truncated 14-3-3-binding motif lacking a proper consensus to a high-affinity site. We notice that the 14-3-3 hydrophobic groove is also used by the *Pseudomonas aeruginosa* toxin ExoS (Ottmann et al., 2007). Both Rnd3 and ExoS peptides lack a proline residue characteristic of other 14-3-3 motifs that allows a sharp exit from the 14-3-3 peptide-binding site (Figure 6C). Instead Rnd3 and ExoS can extend into the 14-3-3 hydrophobic groove. ExoS lacks a phosphoserine and binds 14-3-3 in an opposite orientation (N to C) to Rnd3, but makes analogous contacts on 14-3-3 as the Rnd3 farnesyl group (Figure 6D). ExoS has been assigned as a more specialized mode IV 14-3-3-binding motif, defined by engagement of this 14-3-3 hydrophobic patch (Ottmann et al., 2007). In fact, Rnd3 does not fit either a canonical phospho-Ser/Thr-dependent mode I- or mode II-binding motif for 14-3-3, as it lacks an Arg residue N-terminal to and a Pro residue C-terminal to pSer240 (Figures S2C and 6C) (Obsil and Obsilova, 2011). Without the farnesyl group, Rnd3 would resemble a mode III 14-3-3-binding consensus, matching the sequence pS/pT-X(1-2)-COOH (Figure 6C). Such motifs bind considerably weaker than mode I or mode II (Obsil and Obsilova, 2011). We therefore conclude that Rnd proteins possess a hybrid III/IV 14-3-3-binding motif where the presence of the farnesyl group on the C-terminal Cys241 permits the engagement of the "mode IV" hydrophobic patch on 14-3-3 but retains elements of a consensus resembling the mode III motif (Figures 6B and 6C).

Geranylgeranylated Rnd3 and Rap1A Bind to 14-3-3

The unexpected interaction of the Rnd3 farnesyl group with 14-3-3 led us to investigate the number of isoprenyl groups required for 14-3-3 interaction. We therefore synthesized Rnd3 C-terminal peptides containing between one and four isoprenyl groups (Table 2). Using biolayer interferometry, we obtained similar high-affinity values for the farnesylated and phosphorylated Rnd3 peptide to those obtained by calorimetry (Table 2). We also found that the Rnd3 peptide with pS240 and only one isoprenyl group (C5) had an approximately 10-fold lower affinity for 14-3-3 compared to Rnd3 peptides with two isoprenyl groups (geranyl, C10), three (farnesyl, C15) and four (geranylgeranyl, C20). Interestingly, the affinity gain was mainly achieved by a 5-fold decrease in k_{off} (Table 2). Similarly, Rnd3 peptide pull-down experiments showed that polyisoprenylated phosphopeptides bound most effectively to 14-3-3 (Figure 7A). Together these results indicate that two isoprenyl groups (observed in the crystal structure, Figure 6A) are the minimum requirement for tight 14-3-3 binding, and that the 14-3-3-binding pocket can accommodate the longer geranylgeranyl group as well as the farnesyl moiety.

To determine whether other prenylated proteins in addition to Rnd proteins could interact with 14-3-3 using a hybrid III/IV-binding motif, we searched protein sequence databases using a C-terminal consensus motif, [RK]-X-K-[ST]-C-X(3), defined by key contacts identified in the Rnd3/14-3-3 crystal structure. The search identified the Rnd proteins as well as the geranylgeranylated proteins PDE6C, RPGR, and Rap1A (Figure 7B). Another search was performed using a "relaxed" consensus motif [RK]-X-[RK]-X(0,1)-[ST]-C-X(3), allowing insertion of an extra residue next to the phosphorylated Ser/Thr as well as a less strict requirement for a Lys in the −1/−2 position. Among the candidates, Rap1B was selected because it is closely related to Rap1A (Figure 7B). RhoG was chosen because it has an Arg residue instead of the −1 Lys that directly binds to Asp223 of 14-3-3 (Figures 7B and 6C). Peptides derived from the C termini of these candidate proteins were synthesized with the physiologically relevant prenyl modification and tested for their ability to interact with 14-3-3. Geranylgeranylated and phosphorylated

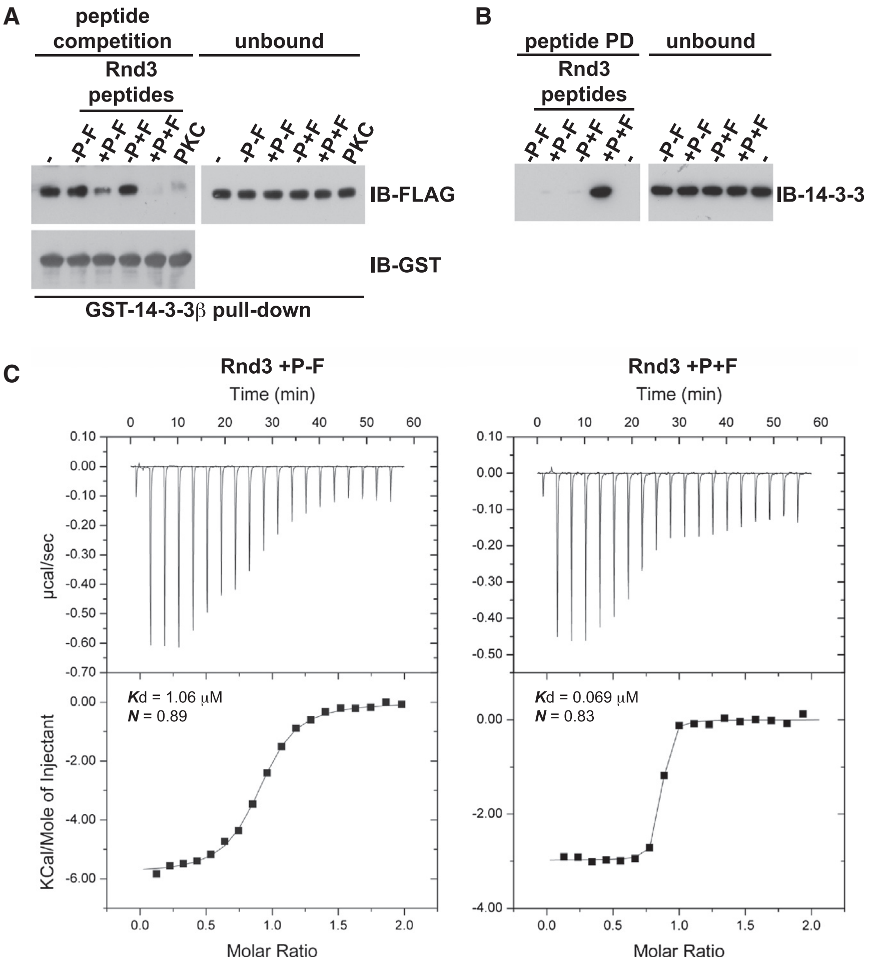

Figure 5. Ser240 Phosphorylation and Cys241 Farnesylation Are Both Essential for Rnd3/14-3-3 Interaction

(A) Farnesylated and/or phosphorylated peptides corresponding to the Rnd3 C terminus (see Table S3) were used as competitors for GST-14-3-3β pull-down experiment with lysates from COS7 cells transfected with FLAG-Rnd3. Bound (peptide competition) and unbound (input) fractions were immunoblotted with the indicated antibodies. A PKCε (PKC) peptide corresponding to a high affinity 14-3-3-binding sequence was used as a positive control.

(B) Biotinylated peptides were incubated with recombinant 14-3-3β, followed by immunoblotting for 14-3-3.

(C) Isothermal titration calorimetry analysis of 14-3-3ζ binding to Rnd3 peptides. Top: raw data; bottom: fitted curves.

−, no peptide; −P−F, no modification; +P−F, with phosphorylation; −P+F, with farnesylation; +P+F, with phosphorylation and farnesylation. See also Figure S5 and Table S3.

Rap1A and PDE6C peptides interacted with 14-3-3 with a similar affinity to the equivalent farnesylated or geranylgeranylated Rnd3 peptides (Tables 2 and S5). RPGR had an intermediate affinity, whereas Rap1B and RhoG peptides displayed a lower affinity for 14-3-3. For RhoG peptide, the low affinity was due to a decrease in k_{on} (Table S5). This suggests that an Arg adjacent to the pSer is poorly tolerated and cannot compensate for Lys at this position. Similarly, the poor Rap1B-binding affinity confirms that Lys at −1 is crucial to the Lys-pSer/Thr-Cys-prenyl motif (Figure 7B).

To validate these data, we pursued the interaction with Rap1A further because its peptide had the highest affinity of those tested for 14-3-3, and it is a Ras superfamily GTPase known to be phosphorylated by PKA on the S180 adjacent to the geranylgeranylated C181 (Quilliam et al., 1991). Biolayer interferometry analysis demonstrated that the phosphorylated and geranylgeranylated peptide displaying an affinity of ~25 nM for 14-3-3ζ, whereas phosphorylated nongeranylgeranylated Rap1A peptide had micromolar affinity (Figure 7C). In addition, Rap1A coimmunoprecipitated with 14-3-3 proteins (Figure 7D). Taken together, these results indicate that the established consensus motif was able to identify new prenyl and pSer-dependent 14-3-3 partners with a hybrid III/IV-binding motif and suggest that additional membrane-anchored proteins could indeed be regulated by 14-3-3.

DISCUSSION

Rnd proteins are atypical G proteins that are not regulated by the classic GDP/GTP switch mechanism. Here we demonstrate they are inhibited by 14-3-3 proteins, which use a novel hybrid III/IV 14-3-3 mode to simultaneously engage the C-terminal farnesyl group and the adjacent phosphorylated serine residues. In addition to Rnd proteins, we find that Rap1A binds in a similar manner to 14-3-3. Here, we report a lipid moiety binding to 14-3-3 and of Ras superfamily GTPases interacting with 14-3-3. Our results also indicate that 14-3-3 proteins act on Rnd proteins similar to the actions of GDIs on other Rho GTPases, by facilitating their extraction from their site of action in membranes and keeping them inactive by sequestration in the cytoplasm. In contrast to GDIs, however, 14-3-3 binding to Rnd proteins requires phosphorylation by upstream protein kinase(s), providing a key regulatory step in their inactivation.

The translocation of Rnd3 from membranes to the cytosol following phosphorylation by ROCK1 or PKC was previously reported (Madigan et al., 2009; Riento et al., 2005). Here we identify that S210 in Rnd3 is the major PKC phosphorylation site, which contributes to 14-3-3 binding and hence loss of membrane localization. In order for PKC activation to induce Rnd3 translocation (Madigan et al., 2009), S240 must have been phosphorylated, as this site is essential for 14-3-3 interaction. ROCK and PKC are members of the AGC kinase family, and most AGC kinases show a preference for phosphorylating Ser/Thr residues downstream of basic residues (Pearce et al., 2010). It is therefore conceivable that other AGC kinases

Table 1. Effect of Rnd3 Peptide Modifications on 14-3-3 Binding

Rnd3 Peptide	Stoichiometry (*N*)	K_d (μM)	TΔS (kcal/mol)	ΔH (kcal/mol)	ΔG (kcal/mol)
+P+F	0.83 ± 0.014	0.069 ± 0.025	6.843 ± 0.24	−2.956 ± 0.06	−9.798 ± 0.23
+P-F	0.89 ± 0.017	1.06 ± 0.04	2.469 ± 0.231	−5.683 ± 0.204	−8.152 ± 0.027
−P+F[a]	-	ND	no binding	-	-
−P-F[a]	-	ND	no binding	-	-

Thermodynamic parameters for Rnd3 C-terminal peptides and 14-3-3ζ-binding constants are shown; mean ± SD, n = 3. Titrations of +P+F and +P-F peptides binding to 14-3-3ζ were carried out at 25°C. ND, not detectable; P, phosphorylation on S240; F, farnesylation; see Table S3 for peptides.
[a]Binding of −P+F and −P-F peptides to 14-3-3ζ was tested at 15°C and 25°C, where binding was not detected.

might phosphorylate Rnd3 and thereby regulate its activity under different conditions.

14-3-3 dimers classically bind to two pSer/pThr residues within the context of surrounding consensus amino acid residues (Obsil and Obsilova, 2011). In Rnd3 none of the three pSer sites contributing to 14-3-3 binding conforms to the consensus mode I or mode II motifs. Our data show that the interaction of the farnesyl moiety with the hydrophobic patch on 14-3-3 strongly increases the affinity of the weak binding of a nonfarnesylated peptide with pS240 to the 14-3-3 phosphate-binding pocket. Moreover, mutational analysis indicates that pS240 is absolutely required for 14-3-3 binding, whereas phosphorylation of S210 and S218 enhances the interaction in cells. It is therefore likely that one of the 14-3-3 subunits in the dimer binds first with high affinity to the farnesyl group and pS240, whereas the other subsequently interacts with either pS210 or pS218. Such combinatorial high- and low-affinity asymmetric interactions have been demonstrated for 14-3-3 interaction with the phosphorylated V3 domain of PKCε (Kostelecky et al., 2009). Based on our results, the previously observed cytosolic translocation induced by PKC phosphorylation of Rnd3 (Madigan et al., 2009) can now be seen as providing the additional 14-3-3 interaction required to complete the extraction from membranes.

Interestingly, we have found that the other Rnd subfamily members, Rnd1 and Rnd2, are also phosphorylated in cells and regulated by 14-3-3 proteins. Like Rnd3, both Rnd1 and Rnd2 require the Ser adjacent to the Cys of the CAAX box and farnesylation to interact with 14-3-3, indicating that they use the hybrid III/IV-binding motif to bind to 14-3-3. Rnd2 has been reported to be mainly cytosolic in NIH 3T3 fibroblasts, although it is also on endosomes (Roberts et al., 2008). The relative level of cytosolic to membrane-bound Rnd proteins is likely to reflect the proportion of the protein that is phosphorylated, which in turn depends on the activity of the upstream kinases. In contrast to Rnd1 and Rnd3, Rnd2 does not induce cell rounding or loss of stress fibers. This could reflect its interaction with a distinct set of proteins (Riou et al., 2010). Rnd2 and Rnd3 act at different stages of cortical neuron migration during development (Pacary et al., 2011), and thus it would be interesting to know how their activity is modulated by the 14-3-3 interaction during this process.

Rnd proteins are part of the Ras superfamily of small GTPases, most of which are posttranslationally modified at the C terminus by farnesylation or geranylgeranylation. We have used a consensus "hybrid III/IV" motif based on the Rnd3 peptide/14-3-3 crystal structure to identify Ras superfamily GTPases as well as other prenylated proteins that might interact with 14-3-3. This allowed us to identify Rap1A as a 14-3-3 partner, implying that Rap1A is likely to be inhibited by phosphorylation and 14-3-3 binding in a similar manner to Rnd proteins. Consistent with this hypothesis, it has been reported that PKA-induced phosphorylation on Ser180 of Rap1A inhibits its interaction with the NADPH oxidase (Bokoch et al., 1991) and Raf (Hu et al., 1999). Another mechanism for extracting Ras and Ras-related GTPases from membranes has been described for PDEδ, which interacts with Ras and Rheb farnesyl groups (Chandra et al., 2012; Ismail et al., 2011; Nancy et al., 2002). We have found that 14-3-3 interaction with Rnd proteins induces their translocation from membranes to the cytosol, but in contrast to RhoGDIs or PDEδ, in this case the process requires Rnd phosphorylation as well as farnesylation.

Most G proteins cycle between an active GTP-bound and inactive GDP-bound form. In contrast, Rnd proteins do not hydrolyse GTP and are constitutively GTP-bound (Riou et al., 2010). We propose that Rnd proteins are instead regulated dynamically by phosphorylation and dephosphorylation. Phosphorylation of Rnd3 C-terminal sites by ROCK and PKC leads to 14-3-3 binding, which then extracts Rnd3 from membranes and prevents it from signaling. ROCKs and most PKCs localize predominantly on membranes when they are active, thereby bringing them in close proximity to Rnd3. The most likely downstream target for Rnd3 on membranes is p190RhoGAP, which leads to downregulation of RhoA and hence loss of stress fibers and cell rounding. Notably, Rnd3 bound to p190RhoGAP is not phosphorylated, supporting our conclusions that Rnd3 is inactivated by phosphorylation and 14-3-3 binding. Conversely, dephosphorylation of Rnd3 by a phosphatase would induce 14-3-3 dissociation, membrane binding, and Rnd3 activation.

Importantly, our results provide a paradigm for regulating prenylated GTP-binding proteins through phosphorylation and subsequent 14-3-3-mediated extraction from membranes by interaction with the farnesyl or geranylgeranyl group. Given that 14-3-3 interaction with Rnd proteins requires the Ser adjacent to the farnesylated Cys, it is not possible for 14-3-3 proteins to bind effectively to this phosphorylated residue without extracting the protein from membranes. Indeed, phosphorylation of Rnd proteins and Rap1A in the cluster of basic amino acids near their C termini is likely to promote membrane extraction by reducing interaction with acidic phospholipids in the

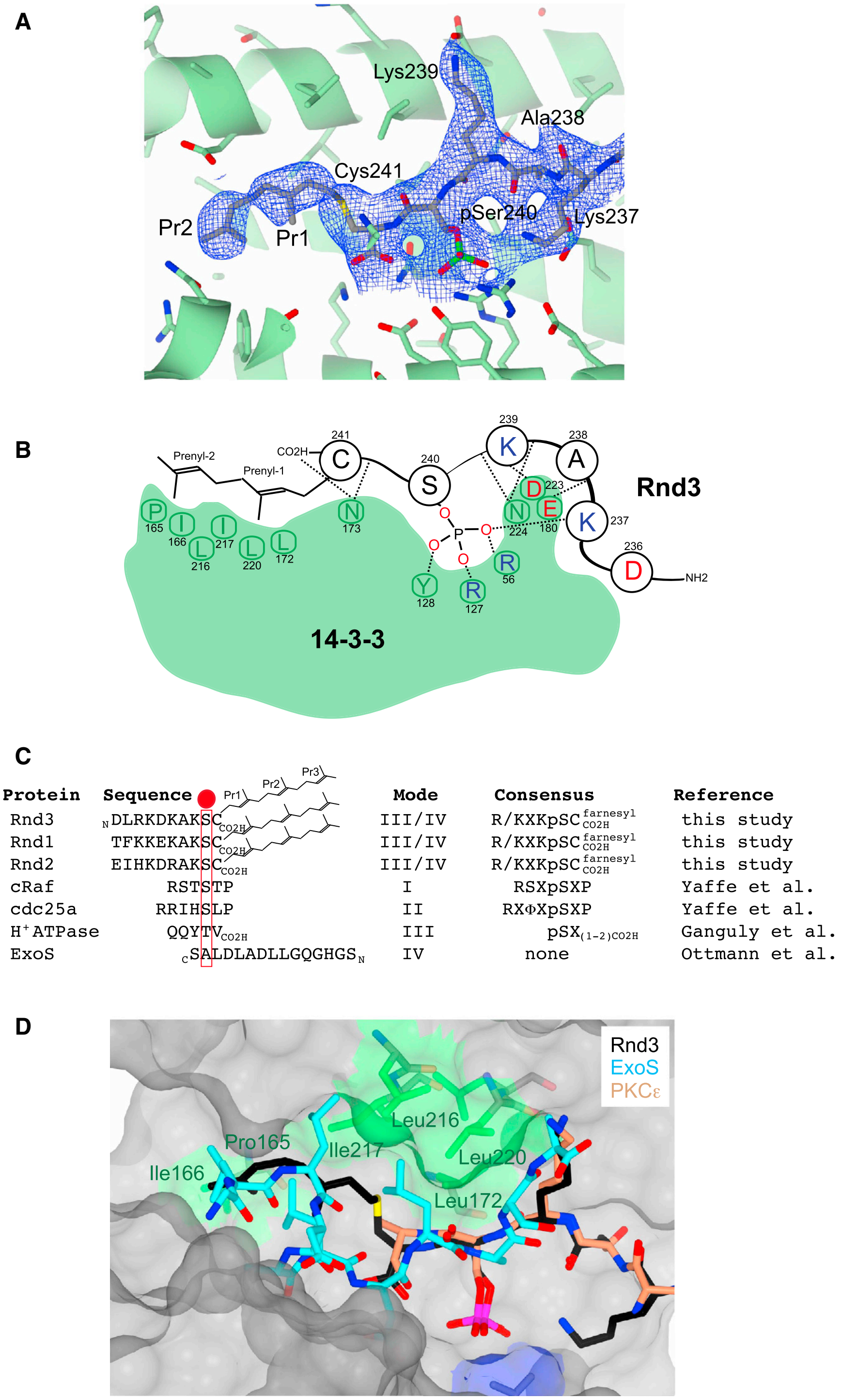

Figure 6. Recognition of Phosphorylated and Farnesylated Rnd3 C-Terminal Peptide by 14-3-3 Reveals a Hybrid III/IV-Binding Mode

(A) Structure of the Rnd3 C-terminal farnesylated phosphopeptide bound to 14-3-3ζ shown with the SIGMAA-weighted $2F_o - F_c$ electron density omit map (σ = 1.0). Electron density is observed for the first two isoprenyl units (Pr1 and Pr2) extending from Cys241.

(B) Schematic view of the Rnd3 C-terminal farnesylated phosphopeptide bound to 14-3-3. Selected side chains from 14-3-3 making contact with Rnd3 are shown.

(C) Sequence alignment of representative 14-3-3-binding motifs indicating how the Rnd family C-terminal motif is a hybrid III/IV motif with features of both a type III and IV motif. Addition of a farnesyl group effectively extends the length of Rnd3 similar to the ExoS-binding interaction. Red ball indicates the phosphoresidue (except for ExoS which has an alanine). The carboxyl terminus of Rnd and H^+ATPase are shown as CO2H and the peptide chain direction of 14-3-3 motif sequences is indicated by an N and C subscript. Note the ExoS peptide is oriented in the opposite direction to all other 14-3-3 motifs (N-C rather than C-N).

(D) Superposition of the Rnd3 C-terminal farnesylated phosphopeptide (black sticks) with the exoenzyme S peptide (ExoS, blue sticks, PDB code 2O02) and PKCε (salmon sticks, PDB code 2WH0), superposed through their respective 14-3-3 partners (gray surface). Hydrophobic residues from 14-3-3 (green sticks and green surface) contacting Rnd3 isoprenyl units 1 and 2 are labeled. Note the ExoS peptide is oriented in an opposing direction (N-C rather than C-N). Atoms are colored according to standard conventions, red, oxygen; blue, nitrogen; yellow, sulfur; gray, carbon; and green, phosphorus. (A) and (D) were prepared using the graphics program PYMOL (http://www.pymol.org).

See also Figure S6 and Table S4.

membrane. This is similar to the proposed "farnesyl-electrostatic switch" mechanism for reduced plasma membrane interaction of K-Ras4B following PKC phosphorylation within its polybasic C terminus (Ahearn et al., 2012). It will be interesting to test if other prenylated proteins identified as 14-3-3 interactors in our peptide screen are also regulated by 14-3-3-dependent extraction from membranes.

EXPERIMENTAL PROCEDURES

Cell Culture and Transfection

COS7 cells grown in Dulbecco's modified Eagle's medium (DMEM) containing 10% bovine fetal calf serum and penicillin/streptomycin (Invitrogen) were electroporated with 5 μg DNA at 250 V and 960 μF using 0.4 cm Gene Pulser Cuvettes (Bio-Rad) in 250 μl of electroporation buffer (120 mM KCl, 10 mM K_2PO_4/KH_2PO_4 [pH 7.6], 25 mM HEPES [pH 7.6], 2 mM $MgCl_2$, and 0.5% Ficoll). Cells were analyzed 24 hr after transfection. NIH 3T3 cells grown in DMEM containing 10% donor bovine serum and penicillin/streptomycin were transfected using FuGENE 6 (Roche) according to the manufacturer's instructions. Where indicated, cells were treated for 20 min with 100 nM calyculin A (Alexis); for 2 hr with 100 nM staurosporine (Calbiochem), 20 μM H-1152 (Calbiochem), 1 μM bisindolylmaleimide I (BIM1, Calbiochem), or 100 nM phorbol-12-myristate-13-acetate (PMA, Cell Signaling Technology); and for 16 hr with 10 μM L-744,832 farnesyltransferase inhibitor (FTI, Calbiochem).

Table 2. Effect of Rnd3 Prenyl Group Length on 14-3-3 Binding

Rnd3 peptide	K_D (nM)	k_{on} (M^{-1} s^{-1})	k_{off} (s^{-1})	χ^2	R^2
−P-F	NB	N/A	N/A	N/A	N/A
+P-F	1300[a]	N/A	N/A	N/A	N/A
+P+Ipr	556	2.4×10^5	1.3×10^{-1}	0.02	0.99
+P+G	56.5	5.7×10^5	3.2×10^{-2}	0.16	0.99
+P+F	32.4	8.6×10^5	2.8×10^{-2}	0.16	0.99
+P+GG	23.2	8.9×10^5	2.1×10^{-2}	0.08	0.99

The affinities of differently modified C-terminal Rnd3 peptides for 14-3-3ζ were determined by biolayer interferometry. Data of a typical experiment (n ≥ 3) along with statistics of the fit (χ^2, R^2) are listed. See Table S3 for Rnd3 peptide sequence. P, phosphorylated on S240; Ipr, isoprenylated; G, geranylated; F, farnesylated; GG: geranylgeranylated; K_D, dissociation constant; k_{on}, association rate constant; k_{off}, dissocation rate constant; NB, no binding; N/A, not applicable.
[a]Determined by steady-state analysis.

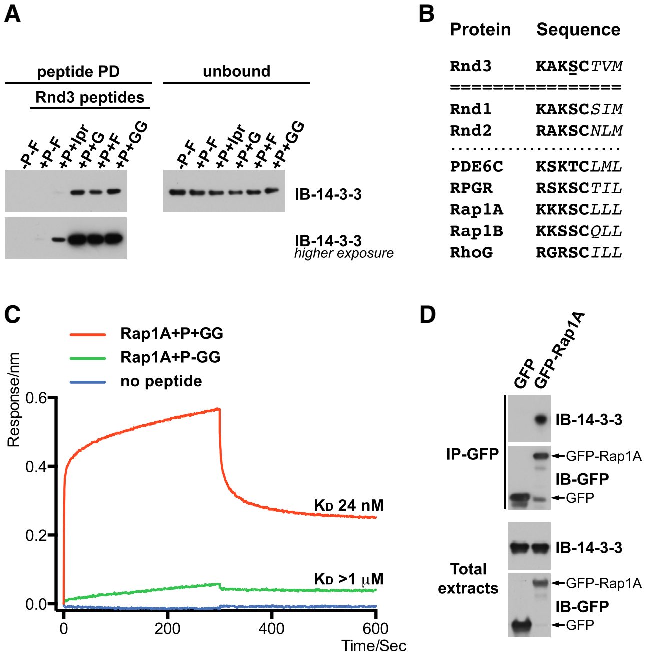

Figure 7. Interaction of Phosphorylated and Prenylated Proteins with 14-3-3

(A) Biotinylated peptides corresponding to the Rnd3 C terminus modified with different prenyl groups were incubated with recombinant 14-3-3ζ, followed by immunoblotting for 14-3-3.

(B) Alignment of C-terminal sequences of prenylated candidate proteins with Rnd3 C-terminal sequence. Sequences are shown in single letter amino acid code; underlined S denotes phosphorylated S240 in Rnd3. The CAAX box residues are represented in italic.

(C) Binding of 14-3-3ζ to immobilized biotinylated Rap1A peptides. Biolayer interferometry was used to assess the binding of a phosphorylated Rap1A peptide with or without the geranylgeranyl modification. The geranylgeranylated peptide has a standard-binding profile. The nonmodified phosphopeptide showed little binding, whereas a negative control with no immobilized peptide gave no response.

(D) Constructs encoding GFP-Rap1A and GFP were transfected into COS7 cells. Endogenous 14-3-3 proteins bound to immunoprecipitated GFP-Rap1A were detected with 14-3-3 antibody. The membrane was probed with the indicated antibodies.

See also Table S5.

Cell Fractionation

Transfected COS7 cells were treated with pharmacological inhibitors as indicated, then washed in cold PBS. Cells were incubated for 5 min in 900 μl of cold fractionation buffer (20 mM Tris HCl [pH 8.0], 150 mM NaCl, 10 mM NaF, 1 mM DTT, 250 mM sucrose) containing EDTA-free protease inhibitor cocktail (Roche) and phosphatase inhibitor cocktails (set II+IV, Calbiochem). Cells were scraped into microfuge tubes and lysed by shearing with a dounce homogenizer. Samples were then centrifuged (4°C, 20 min, 900 *g*). The pellet (nuclear fraction) was washed twice in fractionation buffer, before addition of SDS-Urea loading buffer (8 M Urea, 2% SDS, 50 mM Tris HCl [pH 6.8], 0.3% bromophenol blue). The supernatant fraction was centrifuged (4°C, 60 min, 180,000 *g*) to separate the cytosol (supernatant) from organelles, cytoskeleton and membranes (pellet). Proteins from the cytosol and membrane pellet fractions were precipitated in 10% trichloroacetic acid (4°C, 30 min), centrifuged (13,000 *g*, 10 min), and pellets washed in acetone before resuspension in SDS-Urea loading buffer. Samples were analyzed by SDS-PAGE and immunoblotting.

Immunoprecipitation

Transfected COS7 cells were lysed in lysis buffer LB (1% Triton X-100, 20 mM Tris–HCl [pH 8], 130 mM NaCl, 1 mM dithiothreitol [DTT], 10 mM sodium fluoride, complete EDTA-free protease inhibitor cocktail [Roche], phosphatase inhibitor cocktails [set II+IV, Calbiochem]). After centrifugation (13,000 g, 4°C, 10 min), soluble proteins were precleared then incubated with either mouse nonimmune IgG as a control, mouse anti-HA (HA-7) or mouse anti-FLAG (M2) antibody on agarose beads (Sigma-Aldrich) for 2 hr at 4°C. Beads were washed five times with lysis buffer (containing 260 mM NaCl), then bound proteins were eluted in Laemmli sample buffer (LSB), resolved by SDS–PAGE and analyzed by immunoblotting.

In Vitro Binding Assay with Phosphatase Treatment and In Vitro Kinase Assay

FLAG-Rnd3-transfected COS7 cells were lysed in LB buffer without phosphatase inhibitors, and Rnd3 proteins immunoprecipitated. The beads were washed three times in 0.5 M NaCl, followed by two washes with phosphatase buffer PB (100 mM NaCl, 50 mM Tris-HCl [pH 7.9], 10 mM $MgCl_2$, 1 mM DTT). Beads in PB buffer were separated equally in two tubes and incubated ± calf intestinal alkaline phosphatase (CIP, 20 U; New England Biolabs) at 37°C for 60 min. Equal amount of total lysates from COS7 cells expressing HA-tagged 14-3-3 isoforms were then added to each of the treated beads (±CIP) for 90 min at 4°C for binding. Beads were washed with LB buffer, bound proteins were eluted in LSB, resolved by SDS–PAGE and analyzed by immunoblotting.

Kinase assays were carried out on prewashed beads in kinase buffer (50 mM Tris/HCl [pH 7.5], 10 mM $MgCl_2$, 1 mM DTT, 30 μM ATP, 0.1 μCi/μl [γ -^{32}P]ATP) with 7.5 ng of ROCK1 and PKCζ kinase domains at 30°C for 30 min. Proteins were eluted from beads in LSB, resolved by SDS/PAGE and protein phosphorylation was analyzed by autoradiography of dried gels.

Peptide Pull-Down Assay

Biotinylated peptides (1 to 10 μg; Table S3; Extended Experimental Procedures) were bound to streptavidin-agarose beads (Sigma-Aldrich) for 30 min at 4°C and washed in LB buffer with 0.5% Triton X-100. Recombinant 14-3-3β or ζ (2.5 μg; see Extended Experimental Procedures) was then added and incubated for 60 min at 4°C for binding, followed by five washes prior to elution from beads in LSB, then resolved by SDS–PAGE and analyzed by immunoblotting.

GST-Pull-Down and Peptide Competition Assay

Recombinant GST-14-3-3β was expressed and purified from *E. coli* (Extended Experimental Procedures). FLAG-Rnd3-transfected COS7 cells were lysed in LB buffer. After removal of insoluble material, precleared cell lysates were incubated at 4°C for 2 hr with the recombinant GST or GST-14-3-3β protein (5 μg) on glutathione beads (Amersham Biosciences). Beads were washed

with LB buffer before eluting the proteins in LSB. The proteins were resolved by SDS-PAGE and analyzed by immunoblotting.

For peptide competition experiments (Table S3; Extended Experimental Procedures), peptides (10 μg/ml) were preincubated for 15 min at 4°C with the GST-14-3-3β prior to the addition of precleared cell lysates.

Isothermal Titration Calorimetry and Biolayer Interferometry

ITC measurements were carried out using a VP-ITC200 instrument (GE Healthcare). The 14-3-3ζ domain (see Extended Experimental Procedures) was used at 50–60 μM in ITC buffer (50 mM Tris [pH 7.5]), 100 mM NaCl, 1 mM TCEP). Rnd3 C-terminal peptides were solubilized in ITC buffer and used at 500–600 μM. Titrations were carried out at 25°C in triplicate. Each titration was fitted to a simple one-site binding model using the Origin software provided with the instrument. Titrations with nonbinding peptides were carried out at 15°C and 25°C.

Biolayer interferometry was carried out using an Octet RED96 instrument (ForteBio). Biotinylated peptides were immobilized on streptavadin-coated biosensors (ForteBio) at a concentration of 1 μg/ml in BI buffer (25 mM Tris, 150 mM NaCl, 0.1% Tween-20), for 300 s. The immobilization typically reached a response level of 2 nm. Association and dissociation curves were obtained through addition of a dilution series of 14-3-3ζ (20 to 4,000 μM) for 300 s followed by dissociation in BI buffer for 300 s using the Octet acquisition software. The binding data were fitted using the Octet analysis software.

Crystallization, Data Collection, and Refinement

A peptide corresponding to residues 232-241 of human Rnd3 with Ser-240 phosphorylated and Cys-241 farnesylated (N-term: DLRKDKAKpSC(S-farnesyl); Table S3) was synthesized (Extended Experimental Procedures). The peptide was soluble at 2 mg/ml in buffer A and mixed at a 5-fold molar excess with 14-3-3ζ in buffer A. The 14-3-3ζ:Rnd3 peptide complex was concentrated to 36 mg/ml and crystallized by sitting drop vapor diffusion (well solution: 0.17 M Sodium Acetate; 0.085 M Tris [pH 8.5]; 25.5% (w/v) PEG4000, 15% (v/v) glycerol). Crystallization drops were set up using 200 nl of complex with 200 nl of well solution over 100 μl of well solution. The crystals (with a plate morphology) were flash-frozen in liquid N_2, with cryoprotection provided by the mother liquor.

X-ray diffraction data were collected, on beamline I-24, at the Diamond Light Source. The data were processed and scaled using D*TREK (Pflugrath, 1999). The structure was solved using molecular replacement with PHASER (McCoy et al., 2007) and a 14-3-3 search model derived from the pdb file 2WH0. Refinement, using data to 2.3 Å, was undertaken with PHENIX.REFINE (Afonine et al., 2005) and using torsion angle noncrystallographic symmetry (NCS) and translation/library/screw (TLS) restraints (each 14-3-3 chain having three TLS groups and each Rnd3 peptide a single group) with manual rebuilding using COOT (Emsley and Cowtan, 2004) after each round. Parameters for the Cys-farnesyl link were derived using JLIGAND (Lebedev et al., 2012), with all other parameters coming from the REFMAC monomer library (Vagin et al., 2004).

ACCESSION NUMBERS

The Protein Data Bank accession number for the 14-3-3ζ-Rnd3 peptide atomic coordinates and structure factors reported in this paper is 4BG6.

SUPPLEMENTAL INFORMATION

Supplemental Information includes Extended Experimental Procedures, six figures, and five tables and can be found with this article online at http://dx.doi.org/10.1016/j.cell.2013.03.044.

ACKNOWLEDGMENTS

We thank Michael Yaffe (MIT) for communicating the interaction between 14-3-3ε and Rnd3, providing 14-3-3 constructs, and discussions; Steen Hansen for the p190RhoGAP-B construct; Steve Lynham (Centre for Excellence in Mass Spectrometry, King's College London) for expert mass spectrometry assistance; Gavin Kelly (Cancer Research UK London Research Laboratories) for help with statistical analysis; and Jeroen Claus for designing the graphical abstract. We are grateful to members of the Ridley laboratory for helpful discussions. This work was supported by the BBSRC, MRC, a King's College London Wellcome Trust VIP Award, Cancer Research UK, and the King's College London BHF Centre of Excellence. B.M. was supported by a CJ Martin fellowship.

Received: June 19, 2012
Revised: January 22, 2013
Accepted: March 29, 2013
Published: April 25, 2013

REFERENCES

Afonine, P.V., Grosse-Kunstleve, R.W., and Adams, P.D. (2005). A robust bulk-solvent correction and anisotropic scaling procedure. Acta Crystallogr. D Biol. Crystallogr. *61*, 850–855.

Ahearn, I.M., Haigis, K., Bar-Sagi, D., and Philips, M.R. (2012). Regulating the regulator: post-translational modification of RAS. Nat. Rev. Mol. Cell Biol. *13*, 39–51.

Bokoch, G.M., Quilliam, L.A., Bohl, B.P., Jesaitis, A.J., and Quinn, M.T. (1991). Inhibition of Rap1A binding to cytochrome b558 of NADPH oxidase by phosphorylation of Rap1A. Science *254*, 1794–1796.

Boureux, A., Vignal, E., Faure, S., and Fort, P. (2007). Evolution of the Rho family of ras-like GTPases in eukaryotes. Mol. Biol. Evol. *24*, 203–216.

Chandra, A., Grecco, H.E., Pisupati, V., Perera, D., Cassidy, L., Skoulidis, F., Ismail, S.A., Hedberg, C., Hanzal-Bayer, M., Venkitaraman, A.R., et al. (2012). The GDI-like solubilizing factor PDEδ sustains the spatial organization and signalling of Ras family proteins. Nat. Cell Biol. *14*, 148–158.

Emsley, P., and Cowtan, K. (2004). Coot: model-building tools for molecular graphics. Acta Crystallogr. D Biol. Crystallogr. *60*, 2126–2132.

Forget, M.A., Desrosiers, R.R., Gingras, D., and Béliveau, R. (2002). Phosphorylation states of Cdc42 and RhoA regulate their interactions with Rho GDP dissociation inhibitor and their extraction from biological membranes. Biochem. J. *361*, 243–254.

Foster, R., Hu, K.Q., Lu, Y., Nolan, K.M., Thissen, J., and Settleman, J. (1996). Identification of a novel human Rho protein with unusual properties: GTPase deficiency and in vivo farnesylation. Mol. Cell. Biol. *16*, 2689–2699.

Guasch, R.M., Scambler, P., Jones, G.E., and Ridley, A.J. (1998). RhoE regulates actin cytoskeleton organization and cell migration. Mol. Cell. Biol. *18*, 4761–4771.

Hancock, J.F., Paterson, H., and Marshall, C.J. (1990). A polybasic domain or palmitoylation is required in addition to the CAAX motif to localize p21ras to the plasma membrane. Cell *63*, 133–139.

Hidalgo-Carcedo, C., Hooper, S., Chaudhry, S.I., Williamson, P., Harrington, K., Leitinger, B., and Sahai, E. (2011). Collective cell migration requires suppression of actomyosin at cell-cell contacts mediated by DDR1 and the cell polarity regulators Par3 and Par6. Nat. Cell Biol. *13*, 49–58.

Hoffman, G.R., Nassar, N., and Cerione, R.A. (2000). Structure of the Rho family GTP-binding protein Cdc42 in complex with the multifunctional regulator RhoGDI. Cell *100*, 345–356.

Hu, C.D., Kariya, K., Okada, T., Qi, X., Song, C., and Kataoka, T. (1999). Effect of phosphorylation on activities of Rap1A to interact with Raf-1 and to suppress Ras-dependent Raf-1 activation. J. Biol. Chem. *274*, 48–51.

Ismail, S.A., Chen, Y.X., Rusinova, A., Chandra, A., Bierbaum, M., Gremer, L., Triola, G., Waldmann, H., Bastiaens, P.I., and Wittinghofer, A. (2011). Arl2-GTP and Arl3-GTP regulate a GDI-like transport system for farnesylated cargo. Nat. Chem. Biol. *7*, 942–949.

Kostelecky, B., Saurin, A.T., Purkiss, A., Parker, P.J., and McDonald, N.Q. (2009). Recognition of an intra-chain tandem 14-3-3 binding site within PKCepsilon. EMBO Rep. *10*, 983–989.

Lebedev, A.A., Young, P., Isupov, M.N., Moroz, O.V., Vagin, A.A., and Murshudov, G.N. (2012). JLigand: a graphical tool for the CCP4 template-restraint library. Acta Crystallogr. D Biol. Crystallogr. *68*, 431–440.

Madigan, J.P., Bodemann, B.O., Brady, D.C., Dewar, B.J., Keller, P.J., Leitges, M., Philips, M.R., Ridley, A.J., Der, C.J., and Cox, A.D. (2009). Regulation of Rnd3 localization and function by protein kinase C alpha-mediated phosphorylation. Biochem. J. *424*, 153–161.

McCoy, A.J., Grosse-Kunstleve, R.W., Adams, P.D., Winn, M.D., Storoni, L.C., and Read, R.J. (2007). Phaser crystallographic software. J. Appl. Cryst. *40*, 658–674.

Michaelson, D., Silletti, J., Murphy, G., D'Eustachio, P., Rush, M., and Philips, M.R. (2001). Differential localization of Rho GTPases in live cells: regulation by hypervariable regions and RhoGDI binding. J. Cell Biol. *152*, 111–126.

Morrison, D.K. (2009). The 14-3-3 proteins: integrators of diverse signaling cues that impact cell fate and cancer development. Trends Cell Biol. *19*, 16–23.

Muslin, A.J., Tanner, J.W., Allen, P.M., and Shaw, A.S. (1996). Interaction of 14-3-3 with signaling proteins is mediated by the recognition of phosphoserine. Cell *84*, 889–897.

Nancy, V., Callebaut, I., El Marjou, A., and de Gunzburg, J. (2002). The delta subunit of retinal rod cGMP phosphodiesterase regulates the membrane association of Ras and Rap GTPases. J. Biol. Chem. *277*, 15076–15084.

Nishikawa, K., Toker, A., Johannes, F.J., Songyang, Z., and Cantley, L.C. (1997). Determination of the specific substrate sequence motifs of protein kinase C isozymes. J. Biol. Chem. *272*, 952–960.

Nobes, C.D., Lauritzen, I., Mattei, M.G., Paris, S., Hall, A., and Chardin, P. (1998). A new member of the Rho family, Rnd1, promotes disassembly of actin filament structures and loss of cell adhesion. J. Cell Biol. *141*, 187–197.

Obsil, T., and Obsilova, V. (2011). Structural basis of 14-3-3 protein functions. Semin. Cell Dev. Biol. *22*, 663–672.

Ottmann, C., Yasmin, L., Weyand, M., Veesenmeyer, J.L., Diaz, M.H., Palmer, R.H., Francis, M.S., Hauser, A.R., Wittinghofer, A., and Hallberg, B. (2007). Phosphorylation-independent interaction between 14-3-3 and exoenzyme S: from structure to pathogenesis. EMBO J. *26*, 902–913.

Pacary, E., Heng, J., Azzarelli, R., Riou, P., Castro, D., Lebel-Potter, M., Parras, C., Bell, D.M., Ridley, A.J., Parsons, M., and Guillemot, F. (2011). Proneural transcription factors regulate different steps of cortical neuron migration through Rnd-mediated inhibition of RhoA signaling. Neuron *69*, 1069–1084.

Pearce, L.R., Komander, D., and Alessi, D.R. (2010). The nuts and bolts of AGC protein kinases. Nat. Rev. Mol. Cell Biol. *11*, 9–22.

Pflugrath, J.W. (1999). The finer things in X-ray diffraction data collection. Acta Crystallogr. D Biol. Crystallogr. *55*, 1718–1725.

Quilliam, L.A., Mueller, H., Bohl, B.P., Prossnitz, V., Sklar, L.A., Der, C.J., and Bokoch, G.M. (1991). Rap1A is a substrate for cyclic AMP-dependent protein kinase in human neutrophils. J. Immunol. *147*, 1628–1635.

Riento, K., Totty, N., Villalonga, P., Garg, R., Guasch, R., and Ridley, A.J. (2005). RhoE function is regulated by ROCK I-mediated phosphorylation. EMBO J. *24*, 1170–1180.

Riou, P., Villalonga, P., and Ridley, A.J. (2010). Rnd proteins: multifunctional regulators of the cytoskeleton and cell cycle progression. Bioessays *32*, 986–992.

Roberts, P.J., Mitin, N., Keller, P.J., Chenette, E.J., Madigan, J.P., Currin, R.O., Cox, A.D., Wilson, O., Kirschmeier, P., and Der, C.J. (2008). Rho Family GTPase modification and dependence on CAAX motif-signaled posttranslational modification. J. Biol. Chem. *283*, 25150–25163.

Thorson, J.A., Yu, L.W., Hsu, A.L., Shih, N.Y., Graves, P.R., Tanner, J.W., Allen, P.M., Piwnica-Worms, H., and Shaw, A.S. (1998). 14-3-3 proteins are required for maintenance of Raf-1 phosphorylation and kinase activity. Mol. Cell. Biol. *18*, 5229–5238.

Vagin, A.A., Steiner, R.A., Lebedev, A.A., Potterton, L., McNicholas, S., Long, F., and Murshudov, G.N. (2004). REFMAC5 dictionary: organization of prior chemical knowledge and guidelines for its use. Acta Crystallogr. D Biol. Crystallogr. *60*, 2184–2195.

van Hennik, P.B., ten Klooster, J.P., Halstead, J.R., Voermans, C., Anthony, E.C., Divecha, N., and Hordijk, P.L. (2003). The C-terminal domain of Rac1 contains two motifs that control targeting and signaling specificity. J. Biol. Chem. *278*, 39166–39175.

Wennerberg, K., Forget, M.A., Ellerbroek, S.M., Arthur, W.T., Burridge, K., Settleman, J., Der, C.J., and Hansen, S.H. (2003). Rnd proteins function as RhoA antagonists by activating p190 RhoGAP. Curr. Biol. *13*, 1106–1115.

EXTENDED EXPERIMENTAL PROCEDURES

Antibodies

Rabbit polyclonal antisera were raised against phosphorylated Rnd3-S210 peptide (KNVKRNKpSQRA) by Eurogentec, Seraing, Belgium. Nonspecific antibodies were removed through a nonphosphopeptide (KNVKRNKSQRA) column followed by a specific phosphoenrichment of the antibodies by affinity purification using a phosphopeptide column. The following antibodies were used for immunoblotting and used at 1:1000 dilution, except for the anti-phospho-(Ser) PKC substrate antibody that was used at 1:250 dilution: rabbit polyclonal and mouse monoclonal anti-FLAG (M2, Sigma), rat monoclonal anti-HA antibody (3F10, Roche), rabbit polyclonal anti-phospho-(Ser) PKC substrate antibody (Cell Signaling Technology; broad phospho-Ser motif recognition for AGC kinase substrates), mouse monoclonal anti-14-3-3 (H-8; pan), mouse monoclonal (9E10) and rabbit polyclonal (A-14) anti-Myc antibodies, rabbit polyclonal anti-ERK1/2 antibody, mouse monoclonal anti-GST antibody (B-14), rabbit polyclonal anti-GFP antibody (Santa Cruz), and goat polyclonal anti-CD71 (C-20; TfR) antibody. The following antibodies were used for immunofluorescence: mouse monoclonal anti-FLAG (M2; Sigma) (1:200) and rabbit polyclonal anti-HA (Y-11, Santa Cruz) (1:100).

Plasmids and Mutagenesis

pCMV-FLAG-Rnd3 and pCAG-Myc-ROCK$^{1\text{-}420}$ have been described previously (Riento et al., 2003). pCMV-FLAG-Rnd1 was generated by amplification of human Rnd1 from pRK5-Rnd1 (kindly provided by Catherine Nobes;(Nobes et al., 1998) by PCR and subcloned into the EcoRI and HindIII sites of pCMV-FLAG. pcDNA3-HA-14-3-3β, ε, ζ, σ (Dalal et al., 2004), η and pGEX-4T-14-3-3τ (Bonnefoy-Bérard et al., 1995) and pGEX-4T-14-3-3β (Yaffe et al., 1997) have been described previously. Human 14-3-3-γ cDNA was obtained as an I.M.A.G.E. Consortium clone (Source Bioscience), amplified by PCR and subcloned into the BamHI and XhoI sites of pcDNA3-HA. Mutagenesis of Rnd3 was performed using the QuickChange Site-Directed Mutagenesis Kit (Stratagene) according to the manufacturer's instructions. The nucleotide changes were verified by DNA sequencing (Eurofins MWG operon). pCMV-FLAG-Rnd2 has previously been described (Pacary et al., 2011). pCMV-Myc-Δp190B (encoding amino acids 382-1007 of p190RhoGAP-B) was a gift from Steen Hansen (Wennerberg et al., 2003). pEGFP-Rap1A was a gift from Catherine Hogan (Hogan et al., 2004). Substrate-binding defective 14-3-3 (myc-14-3-3η-R56A/R60A) was a gift from Andrey Shaw (Thorson et al., 1998).

Immunoprecipitation of GFP-Rap1A

COS7 cells transfected with GFP-Rap1A were lysed in 50 mM Tris-Cl pH 7.5, 1 mM EDTA, 1 mM EGTA, 1% Triton X-100, 150 mM NaCl, 270 mM sucrose, 1 mM benzamidine, 0.1% β-mercaptoethanol, 1 mM $NaVO_4$, 50 mM NaF, 1 mM PMSF, 20 nM calyculin A, and protease inhibitor cocktail (Roche). Precleared lysates were added to GFP-Trap coupled to sepharose beads (a kind gift from Carol MacKintosh) for 2 hr. Beads were washed twice with 500 mM NaCl, 50 mM Tris-Cl (pH 7.5), and twice with 50 mM Tris-Cl (pH 7.5), then boiled in Laemmli sample buffer.

Immunoblotting and Phosphodetection

For immunoblotting, proteins from samples in Laemmli sample buffer were separated using precast NuPAGE 4%–12% Bis-Tris gels (Invitrogen), transferred to nitrocellulose membranes (PROTRAN, Whatman), and incubated with antibodies diluted in Tris-buffered saline containing 5% nonfat milk and 0.1% Tween-20 for immunodetection.

For the detection of total level of phosphorylation on immunoprecipitated proteins, proteins were transferred to PVDF membranes (Immobilon-FL, Millipore) and stained using the pro-Q Diamond stain for blot (Molecular Probes) according to the manufacturer's instructions.

Immunofluorescence

Cells were fixed 24 hr after transfection with 4% paraformaldehyde for 20 min, blocked with TBS (25 mM Tris [pH 7.4], 150 mM NaCl) for 10 min, permeabilized for 5 min with PBS containing 0.2% Triton X-100 at 4°C, and incubated at 37°C with antibodies and Alexa fluor phalloidin (wavelength 633 nm; Invitrogen) for F-actin visualization. Specimens were mounted in DAKO fluorescent mounting medium (Dako Cytometrics). Confocal images were acquired with an inverted confocal microscope (LSM 510; Carl Zeiss) using 40× (1.2 NA) or 63× (1.4 NA) objectives. Image analysis was performed using Zen software (Zeiss).

Expression of 14-3-3 Proteins

GST-14-3-3β protein was expressed as previously reported (Yaffe et al., 1997). A hexa-His-tagged version of 14-3-3ζ was expressed and purified essentially as previously reported (Kostelecky et al., 2009). In brief, the *E. coli* strain BL21(DE3) Star (Invitrogen) was transformed with the expression plasmid that was maintained by 34 μg/ml chloramphenicol. *E. coli* cells were grown to OD_{600nm} of 0.7-0.8 in TB medium at 37°C and protein expression induced by 1 mM IPTG for 5 hr at 30°C. Pelleted cells were suspended in buffer A (25 mM Tris [pH 7.5], 100 mM NaCl, 1 mM EDTA, and 1 mM DTT) and disrupted by sonication. Insoluble material was cleared by high-speed centrifugation and the cleared lysate applied to a 5 ml HisTrap column using an AKTA system (GE Healthcare). The 14-3-3ζ protein was eluted with a linear gradient (0–500 mM) of Imidazole in buffer A. Finally, the protein was subjected to

size-exclusion chromatography using a High-Load S200 pg column. As previously described, 14-3-3ζ eluted as a dimer with a molecular weight of ~60 kDa. The final yield of 14-3-3ζ was 30–40 mg per liter of TB medium.

Peptide Synthesis

Solid phase synthesis of peptides was carried out on an ABI 433A peptide synthesizer, with H-Cys(Trt)-ClTrt or Fmoc-Cys(Trt)-TGT resin, using N-Fmoc amino acids and HCTU as the coupling reagent (Merck Chemicals). Each peptide was coupled as follows: Rnd3 (both D double coupled; various peptides ± N-Bio-Eahx), N-DLRKDKAKpSC(S-polyisoprenyl)-C; Rap1A (single coupled), N-Bio-Eahx-EKKKPKKKpSC(S-geranylgeranyl)-C; Rap1B (single coupled), N-Bio-Eahx-PGKARKKSpSC-(S-geranylgeranyl)-C; PDE6C (single coupled), N-Bio-Eahx-GGDDKKSKpTC(S-geranylgeranyl)-C; RPRG (single coupled), N-Bio-Eahx-TNTERRSKpSC(S-geranylgeranyl)-C; RhoG (single coupled), N-Bio-Eahx-PTPIKRGRpSC(S-geranylgeranyl)-C.

Biotin, aminohexanoic acid, and phosphorylated serine/threonine were incorporated in the synthesis as required. Following chain assembly, the peptidyl-resin was added to 10 ml of 92.5% trifluoroacetic acid (TFA), 2.5% ethanedithiol, 2.5% triisopropyl silane, and 2.5% H_2O. After 2 hr, the resin was removed by filtration and peptides were precipitated with diethyl ether on ice. Peptides were isolated by centrifugation, then dissolved in H_2O and freeze-dried overnight. Rnd3 peptide for development of the pseudo-MS^4 assay (see below) was methylated as described in (O'Reilly et al., 2012) with the following additions. The final Fmoc protecting group was left on after chain assembly, then removed after methylation with 20% piperidine in dimethylformamide for 20 min. Following isolation of the phosphorylated methylated peptide after the high acid TFA cleavage, the peptide was treated with a solution of trimethylsilyl bromide (TMSBR; 1.32 ml), ethanedithiol (0.5 ml) m-cresol (0.1 ml) and thioanisole (1.17 ml), cooled to 0°C and allowed to stand for 15 min. The peptide was precipitated with diethyl ether then isolated by centrifugation and freeze drying. This additional step (TMSBr treatment) is required to remove a methyl group from phosphorylated Ser.

Where required, peptides were prenylated with isoprenyl bromide, geranyl bromide, farnesyl bromide (Sigma-Aldrich) or geranylgeranyl chloride (synthesized based on the method in Corey et al., [1972]). Peptides were reacted with 3 equivalents of prenyl bromide/chloride dissolved in methanol and 2 M NaOH at 0°C, pH 10-12 for 15 to 30 min with stirring. Peptides were purified on a C8 reverse phase HPLC column (Agilent PrepHT Zorbax 300SB-C8, 21.2x250 mm, 7 m). Buffer A was 1% acetonitrile, 0.08% trifluoroacetic acid in H_2O, buffer B was 90% acetonitrile, 0.08% trifluoroacetic acid in H_2O. The elution gradient was from 0% to 40% (unprenylated peptides), 5%–40% (isoprenyl), 20%–60% (geranyl and farnesyl), 30%–70% (geranylgeranyl), or 20%–100% (farnesyl methyl) buffer B, over 30 to 40 min at a flow rate of 8 ml/min. The peak fractions were analyzed by LC–MS on an Agilent 1100 LC-MSD. The calculated molecular weights of the peptide were in agreement with the mass found.

Protein Digestion, LC-MS Analysis, and Database Interrogation

Purified immunoprecipitates were eluted in Laemmli sample buffer and separated by SDS-PAGE prior to digestion. In the case of phospho-S240, purified immunoprecipitates were eluted in 5% formic acid and dried. In-gel digestion, LC-MS analysis and database interrogation for phosphorylation analysis of Rnd3 were performed as previously described (Williamson et al., 2010), except that for data interrogation the database taxonomy was restricted to *Homo sapiens*. For characterization of the C-terminal phosphorylated and farnesylated peptide of Rnd3, a pseudo-MS^4 assay was first developed using synthetic peptides of sequence KDKAKSC with and without phospho-Ser and farnesyl and methyl groups on the Cys, predicted to be generated by Arg-C digestion of Rnd3. The dried FLAG-Rnd3 immunoprecipitation eluates were neutralized by reconstituting in 10 μl 1 M triethylammonium bicarbonate (TEAB) and drying *in vacuo* twice. The residue was then reconstituted for digestion in 16 μl of 50 mM TEAB/5% acetonitrile containing 1 μg endoproteinase Arg-C and incubated at 37°C for 4 hr. The reaction was ceased by acidification of the solution to 3% formic acid by the direct addition of neat acid. The reaction vials were then centrifuged at 14,000 g for 2 min and a 5 μl aliquot was extracted from each sample for LC-MS analysis. Reversed phase chromatography was performed using an HP1200 platform (Agilent, Wokingham, UK). Twenty-five percent of each IP sample was analyzed as a 4 μl injection. Peptides were resolved on a 75 μm I.D. 15 cm C18 packed emitter column (3 μm particle size; NIKKYO TECHNOS CO., LTD, Tokyo, Japan) over 30 min using a linear gradient of 96:4 to 30:70 buffer A:B (buffer A: 2% acetonitrile/0.1% formic acid; buffer B: 80% acetonitrile/0.1% formic acid) at 250 nl/min. Peptides were ionized by electrospray ionization using 1.8 kV applied immediately precolumn via a microtee built into the nanospray source. Sample was infused into an LTQ Velos Orbitrap mass spectrometer (Thermo Fisher Scientific, Hemel Hempstead, UK) directly from the end of the tapered tip silica column (6-8 μm tapered tip). The ion transfer tube was heated to 200°C and the S-lens set to 60%. MS^2 and MS^3 scans were acquired using data-dependent acquisition based on a full 30,000 resolution FT-MS scan to sequence the top 6 most intense ions FT-MS using CID fragmentation and 7,500 resolution FT-MS^2 Orbitrap scans with a single repeat count (5 s repeat duration) followed by a 10 s dynamic exclusion with a 10 ppm mass window based on a maximal exclusion list of 500 entries. Automatic gain control was set to 1,000,000 for FT-MS and 50,000 for FT-MS^2, full FT-MS maximum inject time was 500 ms and normalized collision energy was set to 35% with an activation time of 10 ms. MS^3 was also programmed (via a product mass inclusion list) for specific MS^2 product ions 388.21018, 437.19863, 775.41309 and 873.38998 *m/z* corresponding to mass loss of farnesyl, or farnesyl and phosphate, and loss of a single charge from the parent peptide ion, if observed in the top 3 most intense fragment ions. Multistage activation (MSA) was used for both MS^2 and MS^3 scans to cofragment neutral loss of 48.98845, 97.97690, 102.09390 and 151.08235 *m/z*, corresponding to combinatorial neutral losses of phosphate and/or farnesyl, also if observed in the top 3 most intense fragment ions. Data were analyzed manually by direct comparison of the extracted ion chromatograms and inspection of the MS spectra of the digested samples with the synthetic peptide results. This was

performed because the fragmentation profiles of the modified peptides do not score well using traditional proteomic database searching algorithms (data not shown).

SUPPLEMENTAL REFERENCES

Bonnefoy-Bérard, N., Liu, Y.C., von Willebrand, M., Sung, A., Elly, C., Mustelin, T., Yoshida, H., Ishizaka, K., and Altman, A. (1995). Inhibition of phosphatidylinositol 3-kinase activity by association with 14-3-3 proteins in T cells. Proc. Natl. Acad. Sci. USA *92*, 10142–10146.

Corey, E., Takeda, M., and Kim, C. (1972). Method for selective conversion of allylic and benzylic alcohols to halides under neutral conditions. Tetrahedron Lett. *13*, 4339–4342.

Dalal, S.N., Yaffe, M.B., and DeCaprio, J.A. (2004). 14-3-3 family members act coordinately to regulate mitotic progression. Cell Cycle *3*, 672–677.

Hogan, C., Serpente, N., Cogram, P., Hosking, C.R., Bialucha, C.U., Feller, S.M., Braga, V.M., Birchmeier, W., and Fujita, Y. (2004). Rap1 regulates the formation of E-cadherin-based cell-cell contacts. Mol. Cell. Biol. *24*, 6690–6700.

O'Reilly, N., Charbin, A., Lopez-Serra, L., and Uhlmann, F. (2012). Facile synthesis of budding yeast a-factor and its use to synchronize cells of α mating type. Yeast *29*, 233–240.

Riento, K., Guasch, R.M., Garg, R., Jin, B., and Ridley, A.J. (2003). RhoE binds to ROCK I and inhibits downstream signaling. Mol. Cell. Biol. *23*, 4219–4229.

Williamson, R., Thompson, A.J., Abu, M., Hye, A., Usardi, A., Lynham, S., Anderton, B.H., and Hanger, D.P. (2010). Isolation of detergent resistant microdomains from cultured neurons: detergent dependent alterations in protein composition. BMC Neurosci. *11*, 120.

Yaffe, M.B., Rittinger, K., Volinia, S., Caron, P.R., Aitken, A., Leffers, H., Gamblin, S.J., Smerdon, S.J., and Cantley, L.C. (1997). The structural basis for 14-3-3:phosphopeptide binding specificity. Cell *91*, 961–971.

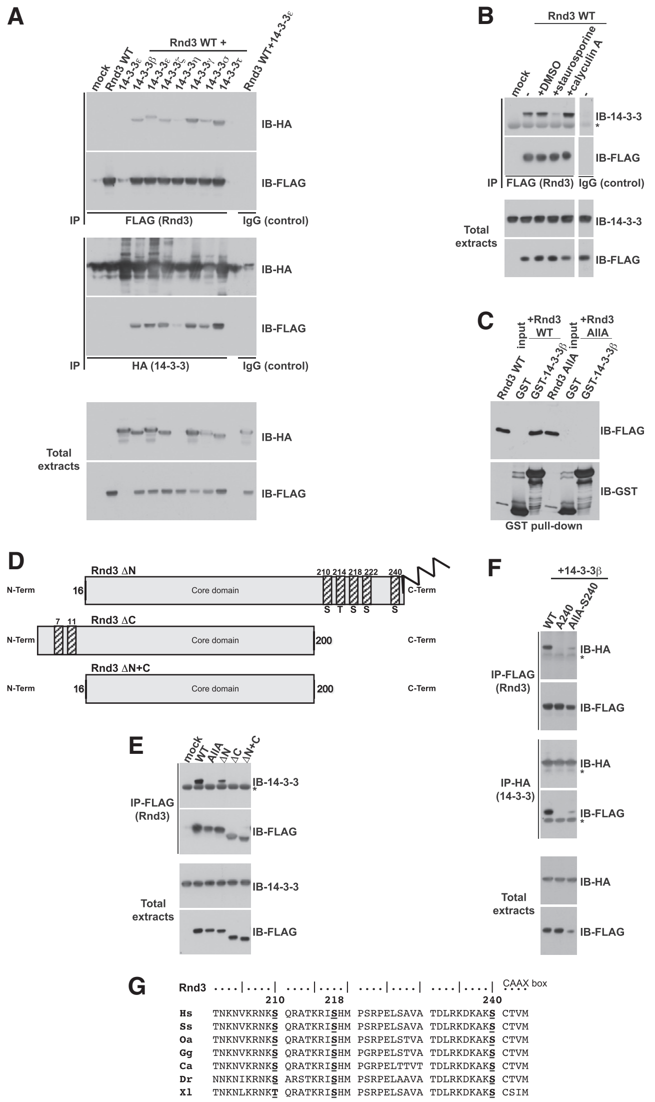

Figure S1. All Mammalian 14-3-3 Isoforms Interact with Rnd3 and C-Terminal Rnd3 Residues Are Required for 14-3-3 Protein Interaction, Related to Figure 1

(A) COS7 cells were cotransfected with wild-type (WT) FLAG-Rnd3 and each of the 7 mammalian HA-14-3-3 isoforms. Cell lysates were immunoprecipitated (IP) with anti-FLAG (top panels), anti-HA (middle panels) antibodies, or nonimmune immunoglobulins (IgG) as a control, then immunoblotted (IB) for HA and FLAG.

(B) Expression vectors encoding FLAG-Rnd3 and HA-14-3-3 isoforms were transfected into COS7 cells. Cells were treated with staurosporine (to inhibit kinases) or calyculin A (to inhibit phosphatases) for 2 hr or 20 min respectively prior to lysis. Cell lysates were immunoprecipitated then western blotted with the indicated antibodies. The space in the gel image marks the position of lanes that were not germane to these results and were thus removed during figure preparation for clarity.

(C) Rnd3-AllA does not bind to 14-3-3 proteins. COS7 cells were transfected with FLAG-Rnd3 or FLAG-Rnd3-AllA. Cell lysates were incubated with GST-14-3-3β or GST as a control, and GST pull-downs were immunoblotted (IB) for GST and FLAG.

(D) Schematic showing 3 Rnd3 deletion mutants.

(E) The indicated FLAG-Rnd3 mutants were transfected into COS7 cells. Cells lysates were immunoprecipitated with anti-FLAG antibody. Endogenous 14-3-3 proteins bound to FLAG-Rnd3 were detected with anti-14-3-3 antibody.

(F) COS7 cells were transfected with the indicated FLAG-Rnd3 constructs and HA-14-3-3β. Cell lysates were immunoprecipitated (IP) with anti-HA or anti-FLAG antibody, then western blotted (IB) for HA and FLAG.

(G) Alignment of Rnd3 C-terminal sequences from representative species. Sequences are shown in single letter amino acid code, with amino acid numbers for *Homo sapiens* (Hs) Rnd3 shown above. Amino acids equivalent to Hs Rnd3 S210, S218 and S240 are underlined. Ss, Sos Scrofus; Gg, Gallus gallus; Dr, Danio rerio; Ca, Crotalus adamanteus; Xl, *Xenopus laevis*; Oa, Ornithorhynchus anatinus. Asterisk (*) indicates IgG light chain.

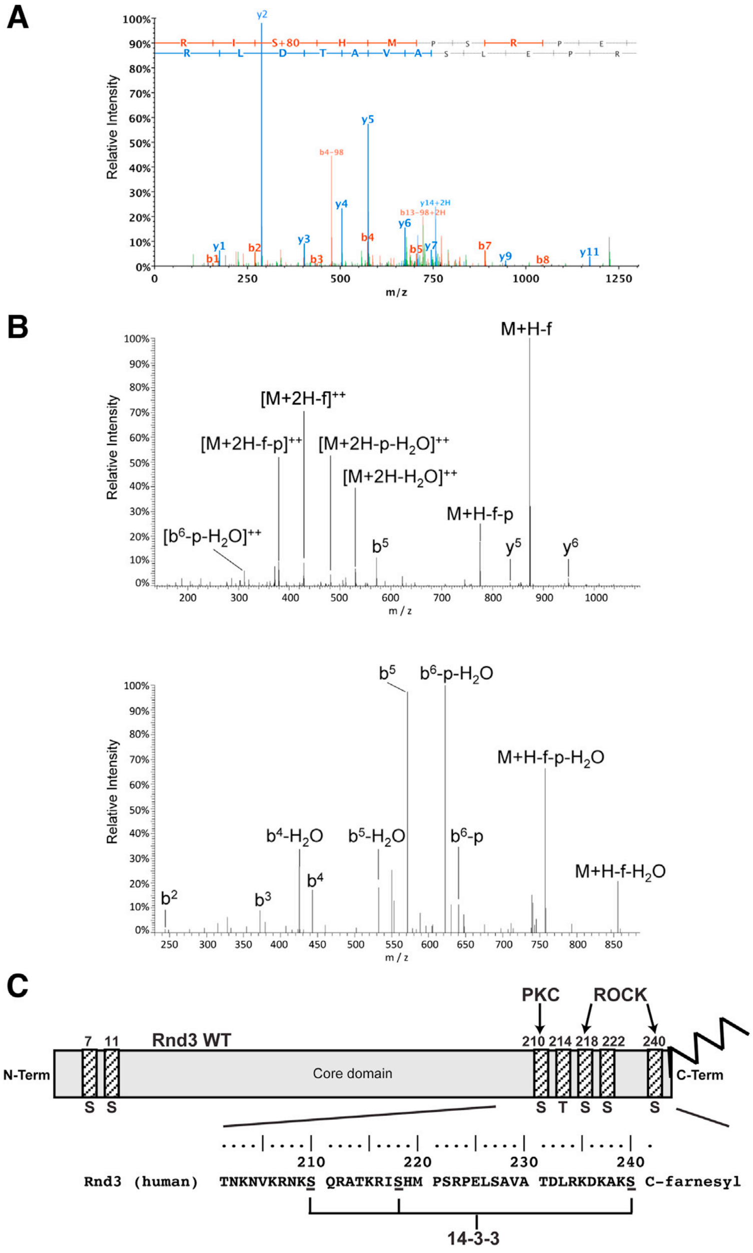

Figure S2. C-Terminal Phosphorylation of Rnd3, Related to Figure 2

(A) Representative MS^2 spectrum indicating phosphorylation of S218 in the sequence ^{216}R-IpSHMPSRPELSAVATDLR-^{234} detected in FLAG immunoprecipitates from COS7 cells cotransfected with FLAG-Rnd3 and myc-ROCK1$^{1\text{-}420}$, or transfected with FLAG-Rnd3 and incubated with calyculin A for 20 min. Precursor *m/z*: 554.79^{4+}, mass error 17 ppm. Data were acquired using an Ultimate LC platform (Dionex, Camberley, UK) coupled online to a QToF-micro mass spectrometer (Waters, Manchester, UK) for data-dependent LC-MS^2.

(B) Representative (i) MS^2 and (ii) MS^3 spectra indicating phosphorylation, farnesylation and carboxymethylation of the C-terminal peptide of Rnd3 KDKAKpSfC-OMe, detected in FLAG immunoprecipitates from COS7 cells cotransfected with FLAG-Rnd3 and myc-ROCK1$^{1\text{-}420}$, or transfected with FLAG-Rnd3 and incubated with calyculin A for 20 min. Precursor ion for MS^2: *m/z* 539.29248^{2+}, mass error −0.1 ppm (30,000 resolution scan); precursor for MS^3: *m/z* 873.38391^{1+}, mass error −6.9 ppm (7,500 resolution scan). Data were acquired using an HP1200 nanoLC platform (Agilent, Wokingham, UK) coupled online to a LTQ Velos Orbitrap mass spectrometer (ThermoFisher Scientific, Hemel Hempstead, UK) for data-dependent LC-MS^2 and LC-MS^3 with all mass measurements performed in the Orbitrap mass analyzer. Annotations are: M – parent peptide mass, f – loss of farnesyl (204.1878 Da) and p – loss of phosphate (97.9769 Da).

(C) Schematic showing Rnd3 C-terminal sequence and phosphorylation sites, indicating the 3 sites involved in 14-3-3 binding and their respective kinases. Sequence is shown in single letter amino acid code, with underlined S denoting phosphorylated serine.

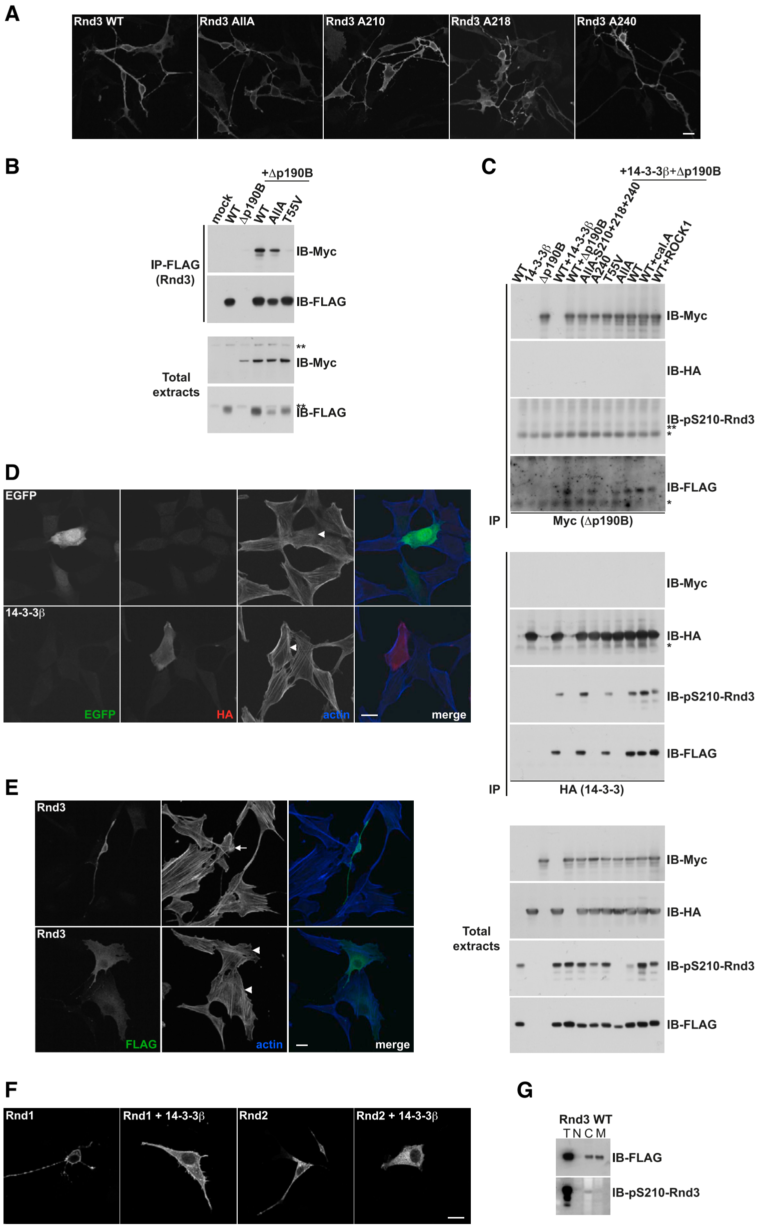

Figure S3. Rnd3 Phosphorylation Is Not Required for Morphological Changes or p190RhoGAP Interaction, Related to Figure 3

(A) Cell morphology and FLAG-Rnd3 localization in NIH 3T3 cells transfected with the indicated constructs. Bar, 20 μm.

(B) The indicated FLAG-Rnd3 constructs and Myc-p190RhoGAP-B deletion mutant (Δp190B) were transfected into COS7 cells. Cell lysates were immunoprecipitated with anti-FLAG antibody. Myc-p190RhoGAP protein bound to FLAG-Rnd3 was detected with anti-Myc antibody.

(C) The indicated FLAG-Rnd3 constructs, HA-14-3-3β and Δp190B were transfected into COS7 cells with or without ROCK1$^{1\text{-}420}$ cotransfection or calyculin A (cal. A) treatment where indicated. Cell lysates were immunoprecipitated with anti-Myc (upper panels) or anti-HA (middle panels) antibodies followed by immunoblotting with the indicated antibodies.

(D–F) NIH 3T3 cells were transfected with the indicated constructs. (D) Single confocal images (maximum intensity z stack projections shown in merge) illustrate cell morphology together with GFP and HA (14-3-3β) localization. Arrowheads (normal phenotype) indicate GFP and HA-14-3-3β-expressing cells (F-actin images). (E) Cell morphology together with FLAG (Rnd3) and F-actin localization. Cells transfected with wild-type Rnd3 show the typical Rnd3-induced rounded phenotype with long thin protrusions (upper panels, arrow), whereas some cells have a distinct phenotype (lower panels, arrowheads). (F) Confocal images of cells expressing FLAG-Rnd1 or FLAG-Rnd2 with or without HA-14-3-3β, and stained for FLAG. Bars, 20 μm.

(G) FLAG-Rnd3 WT was transfected into COS7 cells, prior to cell lysis and biochemical fractionation. The different fractions were immunoblotted with the indicated antibodies. T: Total extracts; N: Nuclear fraction; C: Cytosolic fraction; M: Membrane fraction. Asterisk (*) indicates IgG light chain, double asterisk (**) indicates a nonspecific band.

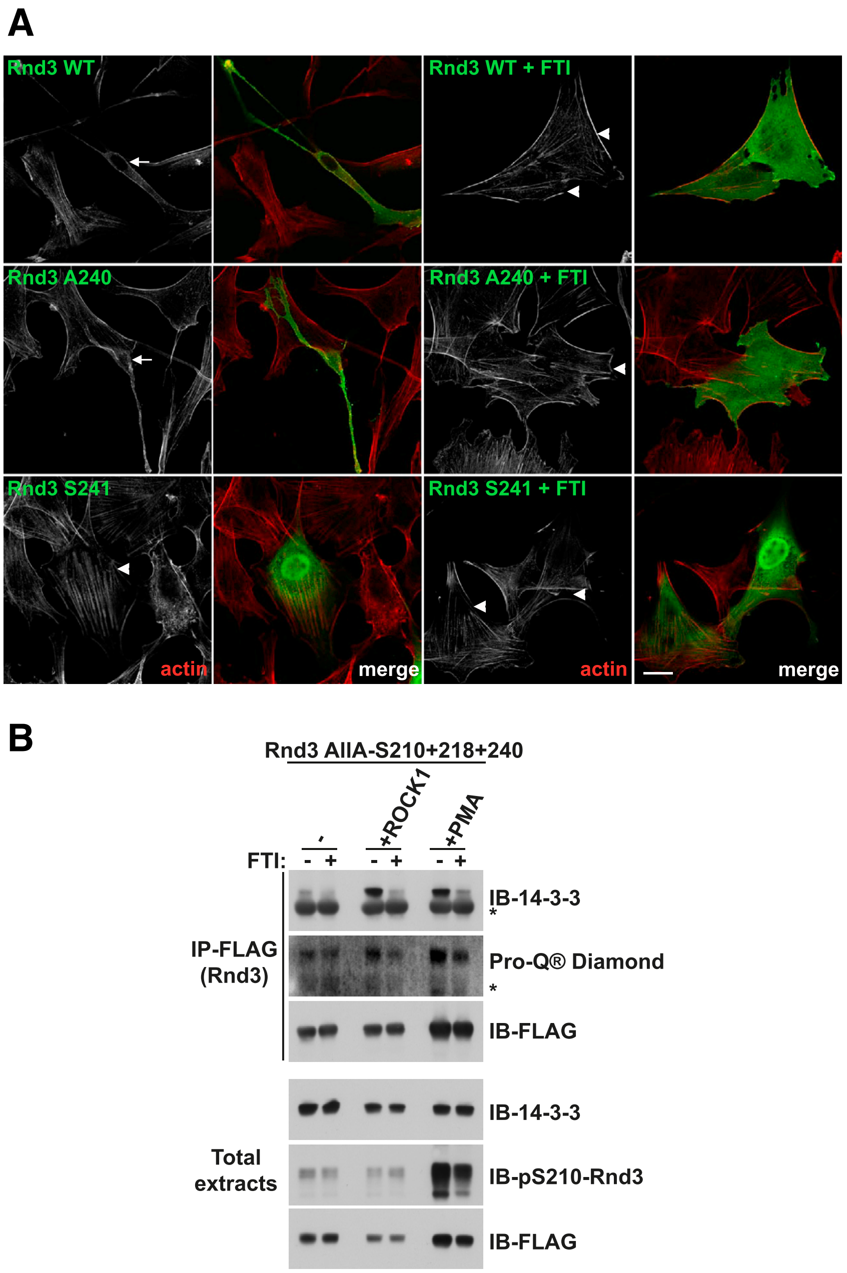

Figure S4. Farnesylation Is Required for Rnd3 Binding to 14-3-3 Proteins, Related to Figure 4

(A) Cell morphology together with FLAG (Rnd3) and F-actin localization in NIH 3T3 cells transfected with the indicated Rnd3 constructs and treated with FTI or DMSO as a control for 16-18 hr. Bar, 20 μm.

(B) FLAG-Rnd3-AllA-S210+218+240 mutant ± ROCK1$^{1-420}$ were transfected into COS7 cells. Cells were treated ± FTI for 16–18 hr and PMA for 2 hr where indicated prior to cell lysis. Cell lysates were immunoprecipitated with anti-FLAG antibody. Endogenous 14-3-3 proteins bound to FLAG-Rnd3 were detected with anti-14-3-3 antibody. Membranes were also stained with Pro-Q Diamond reagent or immunoblotted with the indicated antibodies. Asterisk (*) indicates light chain IgG.

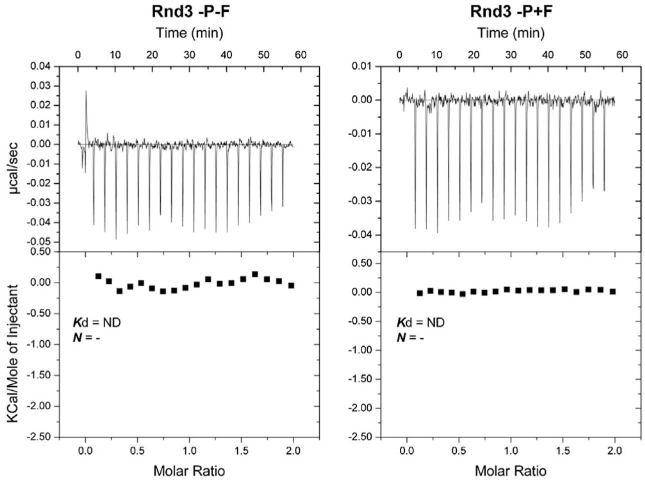

Figure S5. Rnd3 Farnesylation and Phosphorylation Are Required for Interaction with 14-3-3 Proteins, Related to Figure 5

Isothermal titration calorimetry analysis showing the absence of 14-3-3ζ binding to unphosphorylated Rnd3 peptides with or without farnesylation. Raw data are shown in the top panels with fitted curves below. −P-F: no modification; −P+F: with farnesylation; ND: not detectable.

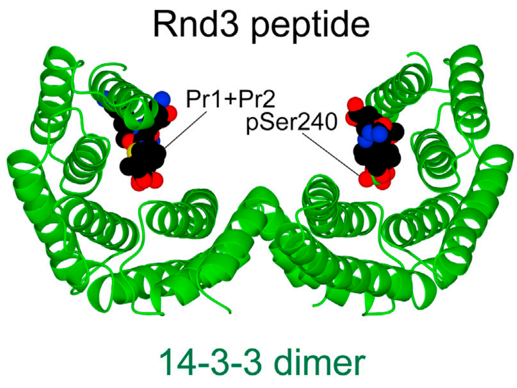

Figure S6. Structure of Rnd3 C-Terminal Peptide with 14-3-3ζ, Related to Figure 6

Crystal structure of 14-3-3ζ dimer (green ribbon) with one Rnd3 phosphorylated and farnesylated peptide bound to each 14-3-3 monomer (solid rendering, colored by atom type). The two-fold axis of the 14-3-3 dimer is vertical in this view ensuring that each Rnd3 peptide traverses C-N or N-C toward the viewer presenting either the prenyl moieties (Pr1 + Pr2) and Cys241 (left hand peptide, black and yellow) or pSer240 (right hand peptide, red), both are labeled for clarity.
